# Mesenchymal stem cell‐mediated mitochondrial transfer regulates the fate of B lymphocytes

**DOI:** 10.1111/eci.70073

**Published:** 2025-05-15

**Authors:** Veronika Somova, Natalie Jaborova, Bianka Porubska, Daniel Vasek, Natalie Fikarova, Martin Prevorovsky, Zuzana Nahacka, Jiri Neuzil, Magdalena Krulova

**Affiliations:** ^1^ Department of Cell Biology, Faculty of Science Charles University Prague Czech Republic; ^2^ Laboratory of Molecular Therapy Institute of Biotechnology, Czech Academy of Sciences Prague‐West Czech Republic; ^3^ Department of Physiology, Faculty of Science Charles University Prague Czech Republic; ^4^ School of Pharmacy and Medical Science Griffith University Southport Queensland Australia; ^5^ First Faculty of Medicine Charles University Prague Czech Republic

**Keywords:** B cell, immunoregulation, mesenchymal stem cell, metabolism, mitochondria

## Abstract

**Background:**

Mitochondrial transfer is becoming recognized as an important immunomodulatory mechanism used by mesenchymal stem cells (MSCs) to influence immune cells. While effects on T cells and macrophages have been documented, the influence on B cells remains unexplored. This study investigates the modulation of B lymphocyte fate by MSC‐mediated mitochondrial transfer.

**Methods:**

MSCs labelled with MitoTracker dyes or derived from mito::mKate2 transgenic mice were co‐cultured with splenocytes. Flow cytometry assessed mitochondrial transfer, reactive oxygen species (ROS) levels, apoptosis and mitophagy. Glucose uptake was measured using the 2‐NBDG assay. RNA sequencing analysed gene expression changes in CD19+ mitochondria recipients and nonrecipients. Pathway analysis identified affected processes. In an LPS‐induced inflammation model, mito::mKate2 MSCs were administered, and B cells from different organs were analysed for mitochondrial uptake and phenotypic changes. MSC‐derived mitochondria were also isolated to confirm uptake by FACS‐sorted CD19+ cells.

**Results:**

MSCs transferred mitochondria to CD19+ cells, though less than to other immune cells. Transfer correlated with ROS levels and mitophagy induction. Mitochondria were preferentially acquired by activated B cells, as indicated by increased CD69 expression and glycolytic activity. Bidirectional transfer occurred, with immune cells exchanging dysfunctional mitochondria for functional ones. CD19+ recipients exhibited increased viability, proliferation and altered gene expression, with upregulated cell division genes and downregulated antigen presentation genes. In vivo, mitochondrial acquisition reduced B cell activation and inflammatory cytokine production. Pre‐sorted B cells also acquired isolated mitochondria, exhibiting a similar anti‐inflammatory phenotype.

**Conclusions:**

These findings highlight mitochondrial trafficking as a key MSC‐immune cell interaction mechanism with immunomodulatory therapeutic potential.

## Graphical abstract

Mesenchymal stem cells (MSCs) modulate immune responses by transferring mitochondria to immune cells, including B lymphocytes. This study demonstrates that mitochondrial transfer to CD19+ cells is linked to reactive oxygen species levels, mitophagy and activation. Mitochondria acquisition enhances B cell viability and proliferation while downregulating antigen presentation. In vivo, mitochondrial uptake reduces B cell activation and inflammatory cytokine production. These findings highlight mitochondrial trafficking as a key immunoregulatory mechanism with potential therapeutic implications in inflammation and immune modulation.

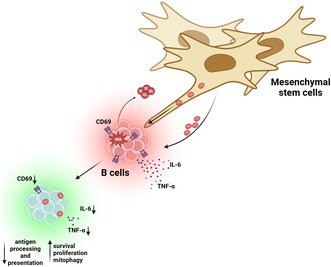

## 1 INTRODUCTION

The therapeutic potential of mesenchymal stem/stromal cells (MSCs) has been attributed to a complex network of mechanisms that modulate the immune environment, with paracrine secretion of soluble factors playing an important role. However, several recent studies have shown that intercellular (horizontal) mitochondrial transfer is one of the essential mechanisms by which MSCs stimulate regeneration and reduce inflammation by balancing cellular stress in various cell types.^1^, ^2^

Mitochondria play a key role in many biological processes, such as aerobic metabolism, calcium signalling and cell death pathways, and current trends highlight the importance of mitochondria in health and disease. Defects in mitochondrial function are associated with many human pathologies, including lung disease, diabetes, vascular disease and various degenerative diseases.^3^, ^4^, ^5^ Transfer of healthy mitochondria has been considered as an effective therapeutic strategy. However, its substantial potential is often overlooked. MSCs have high capacity for ex vivo expansion and possess unique regenerative capabilities, including an immune‐privileged status that allows their use in an allogeneic setting. Given their immunomodulatory properties and the ability to migrate to the site of injury, they are the most studied cells known to donate mitochondria.

The signals that trigger mitochondrial transfer from MSCs are not yet well understood. On the other hand, it is known that primarily stress signals such as ROS, CD73‐generated adenosine, phosphatidylserine on the surface of apoptotic cells and the release of damage‐associated molecular patterns (DAMPs) can induce mitochondrial transfer to cells ‘in need’.^4^, ^6^, ^7^ Mitochondrial transfer can be mediated by various mechanisms that include tunnelling nanotube, gap junctions, extracellular vesicles (EVs) and cell fusion, and occurs under both physiological and pathological conditions.^1^, ^8^ Although mitochondrial transfer is generally associated with the rescue of aerobic respiration and tissue regeneration, its negative effects on the organism have been described in case of cancer.^9^

Few studies have focused on mitochondrial transfer from MSCs to immune cells. There are in vitro and in vivo studies that have shown transferred mitochondria to induce selective differentiation of macrophages into the M2 type. Furthermore, the overall bioenergetic status and phagocytic and antimicrobial capacities of mitochondrial recipients were significantly improved compared to controls.^10^, ^11^, ^12^ Acquisition of mitochondria also promoted expression of genes associated with the regulatory phenotype of T lymphocytes, including *FOXP3, IL2RA* and *CTLA4*, and improved the overall immunosuppressive capacity of Treg cells.^13^, ^14^, ^15^ Furthermore, co‐culture of MSCs with Th17 cells significantly impaired IL‐17 production, and, accordingly, MSCs isolated from patients with rheumatoid arthritis showed impaired mitochondrial transfer to Th17 cells compared to healthy MSCs.^16^ Mitochondrial transfer to activated Th cells after co‐culture with MSCs resulted in downregulation of T‐bet, which is the master Th1 transcription factor, IFN‐γ production and subsequent Th1 effector functions.^17^

These data demonstrate that mitochondrial transfer from MSCs to immune cells plays an important role in immunosuppression; hence, it may represent a novel target for MSC‐mediated therapies and could be used to facilitate immune responses. Therefore, the objective of this study was to investigate signals that trigger mitochondrial transfer from MSCs to immune cells and to evaluate its impact on the phenotype of recipient cells, with particular emphasis on B cells. B cells were chosen as a relevant model for studying the effects of mitochondrial transfer as they receive fewer mitochondria than other immune cell populations.^16^

## 2 RESULTS

### 2.1 MSC‐mediated mitochondrial transfer has a different impact on individual leukocyte populations

**FIGURE 1:**
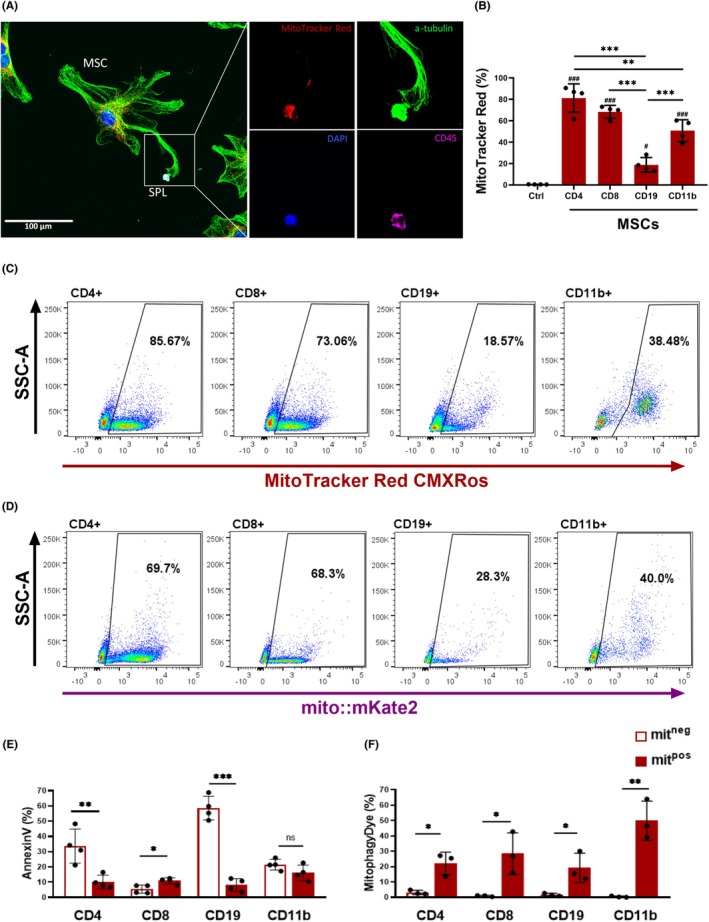
MSC‐mediated mitochondrial transfer to different immune cell populations. Splenocytes were co‐cultured with MitoTracker Red‐stained MSCs or with MSCs isolated from mito::mKate2 mice for 3 h and the positivity of MitoTracker or mKate2 fluorescence, expression of Annexin V or mitophagy of recipient cells was evaluated by flow cytometry. (A) Representative immunofluorescence image of mitochondrial transfer from MSC to CD45^+^ cell MitoTracker Red (red), α‐tubulin (green), CD45 (magenta), nuclei stained with DAPI (blue). (B) Quantification of mitochondrial transfer to different immune cell populations shown as a percentage of MitoTracker Red‐positive cells. MSCs—mitochondrial transfer to splenocytes co‐cultured with MitoTracker Red‐stained MSCs. Ctrl—control immune cells cultured without MSCs. Data are presented as mean ± SD of four independent experiments. (C, D) Representative dot plots illustrating the extent of mitochondrial transfer into individual immune cell populations. (C) Dot plots of populations that acquired mitochondria from MitoTracker Red labelled MSCs. (D) Dot plots of populations that acquired mitochondria from mito::mKate2 positive MSCs. (E) Quantification of apoptotic cells shown as a percentage of Annexin V+ mit^neg^ and mit^pos^ leukocyte populations. Data are expressed as mean ± SD of four independent experiments. (F) Quantification of mitophagy shown as a percentage of mit^neg^ and mit^pos^ immune cell positive for MitophagyDye. Data are presented as mean ± SD of three independent experiments (*n* = 3), where *n* corresponds to the number of mice used. In all figures, statistically significant differences between groups are indicated by asterisks (*p < .05, **p < .01 and ***p < .001), hashtags show statistically significant differences between groups and Ctrl (^#^ *p* < .05, ^##^ *p <* .01 and ^###^ *p <* .001).

To determine whether MSCs transfer mitochondria to primary leukocytes, we labelled MSCs with the MitoTracker Red probe and co‐cultured them with syngeneic splenocytes for 3 h. As shown by microscopy, MSCs transfer mitochondria to CD45^+^ leukocytes (Figure 1A). This finding was confirmed by flow cytometry analysis, which revealed different extents of mitochondrial transfer into the studied populations that included CD4^+^ T cells, CD8^+^ T cells, CD19^+^ B cells and CD11b^+^ myeloid cell populations. The highest mitochondrial uptake was observed in T cells, while the lowest uptake was observed in CD19^+^ cells (Figure 1B,C). To eliminate the possibility of passive staining of immune cell mitochondria by the MitoTracker probe leak, we evaluated mitochondrial transfer from MSCs obtained from mito::mKate2+ mice. We observed no significant difference in the percentage of immune cells that acquired mitochondria in both allogeneic (mito::mKate2+ MSCs to BALB/c‐derived immune cells) and syngeneic (mito::mKate2+ MSCs to B6‐derived immune cells—data not shown) settings (Figure 1D). This suggests that mitochondrial transfer is a mechanism that functions across MHC differences. To monitor the fate of cells that acquired or did not acquire mitochondria, we investigated the apoptosis levels using Annexin V, mitochondrial recipients (mit^pos^) and nonrecipients (mit^neg^) in individual cell populations. In particular, the results showed that CD4^+^ and CD19^+^ mit^pos^ cells had a lower level of phosphatidylserine after mitochondrial uptake compared to mit^neg^ cells. In contrast, CD8^+^ mit^pos^ cells were more susceptible to apoptosis (Figure 1E).

Since autophagy is an important regulator of leukocyte function,^18^ we investigated whether mitochondrial uptake triggers this process. Mitophagy was only detected in a very small percentage of mit^neg^ cells, whereas approximately 20%–30% of mit^pos^ CD4^+^, CD8^+^ and CD19^+^ cells and 50% of CD11b^+^ cells were positive for mitophagy‐specific staining (Figure 1F). In the Transwell system where MSCs and splenocytes were separated by a membrane, mitochondrial transfer was completely abolished (Figure S1). This suggests that the transfer occurs through a contact‐dependent mechanism.

### 2.2 MSC‐mediated mitochondrial transfer correlated with ROS levels in CD4 ^+^ and CD19 ^+^ cells

**FIGURE 2:**
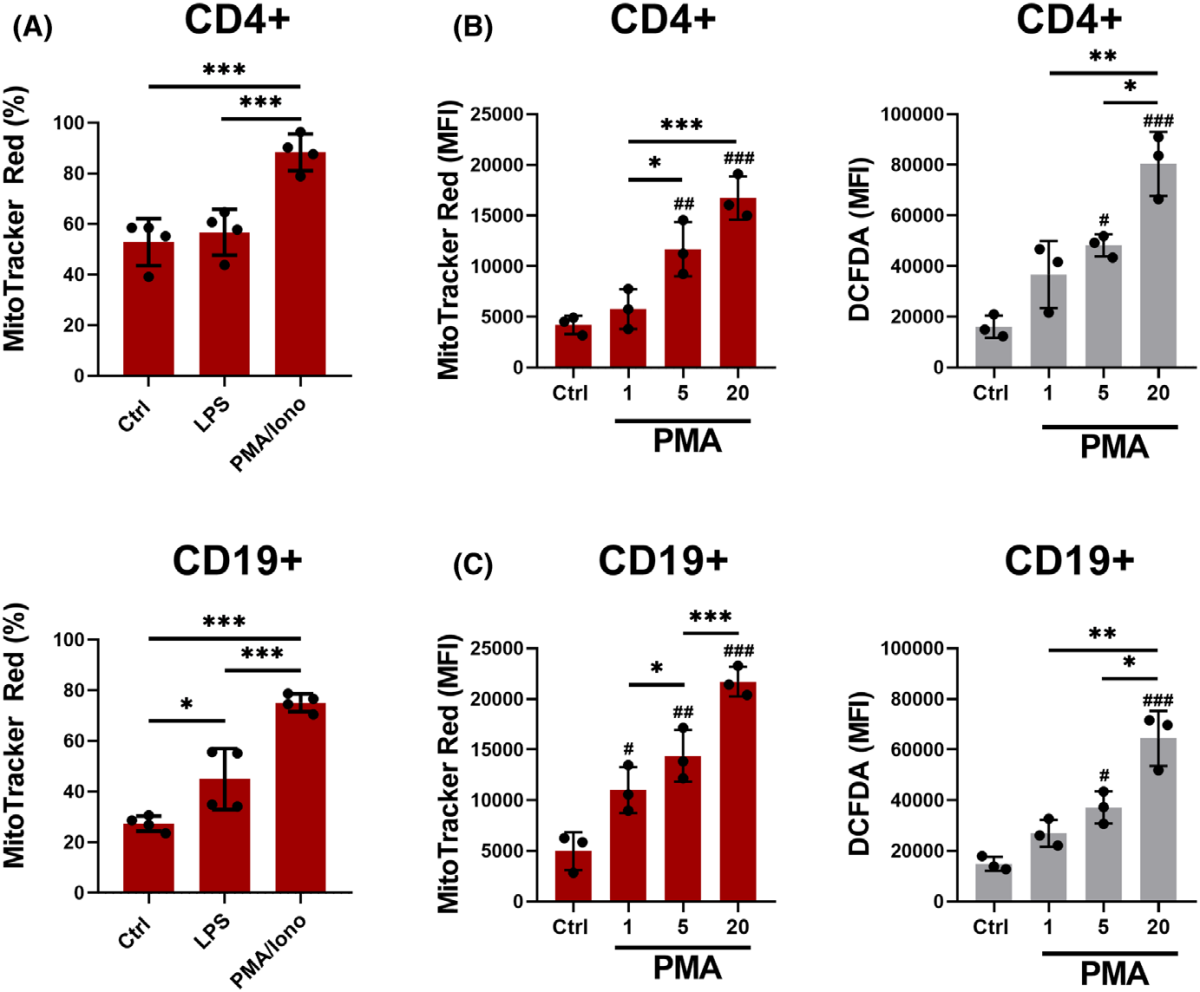
Induction of ROS production and its effect on mitochondrial transfer. (A) The effect of 1.25 μg/mL LPS or 20 ng/mL PMA and 1 μg/mL Ionomycin treatment on the efficacy of mitochondrial transfer. Immune cells were left unstimulated (Ctrl) or treated with either lipopolysaccharide (LPS) or the combination of PMA and Ionomycin (PMA/Iono) for 24 h before co‐culture with MitoTracker Red‐stained MSCs. Data are presented as mean ± SD of four independent experiments. (B, C) The effect of PMA treatment dosing (1/5/20 ng/mL) with fixed concentration of Ionomycin at 1 μg/mL on the efficacy of mitochondrial transfer to CD4+ (B) and CD19+ (C) and corresponding ROS production shown as mean fluorescence (MFI) of DCFDA (B, C). Data are presented as mean ± SD of three independent experiments (*n* = 3), where *n* corresponds to the number of mice used. In all figures, statistically significant differences between groups are indicated by asterisks (*p < .05, **p < .01 and ***p < .001), hashtags show statistically significant differences between groups and Ctrl (^#^ *p* < .05, ^##^ *p <* .01 and ^###^ *p <* .001).

These findings highlight the diverse effects of mitochondrial transfer on leukocyte populations, including apoptosis and mitophagy, and suggest that this process is mediated through direct cell contact. Building upon this, we aimed to investigate the role of reactive oxygen species (ROS) as a potential regulator of mitochondrial transfer, focusing specifically on CD4+ and CD19+ cells due to their contrasting abilities to acquire mitochondria. To induce MSC‐mediated mitochondrial transfer, splenocytes were stimulated with either LPS or PMA/ionomycin, both known to induce ROS.,^19^, ^20^ as previous studies have indicated that ROS levels could play a role in mitochondrial transfer.^18^, ^21^ The percentage of CD4^+^ cells that were positive for MitoTracker increased significantly when stimulated with PMA/ionomycin. Similarly, CD19^+^ cells exhibited higher mitochondrial acquisition when stimulated with LPS or PMA/ionomycin (Figure 2A). Moreover, a correlation was observed between mitochondrial transfer efficacy (as indicated by MitoTracker intensity) and ROS levels in both populations (Figure 2B,C). In an additional study, we noted that mitochondrial transfer could be suppressed when acceptor cells were pre‐treated with antioxidants such as NAC. Conversely, treatment with H_2_O_2_ significantly increased mitophagy, potentially promoting mitochondrial uptake (Figure S4).

### 2.3 Activation, glycolytic activity and complex regulation involving protein trafficking inhibitors and antioxidants influence MSC‐mediated mitochondrial transfer to immune cells

**FIGURE 3:**
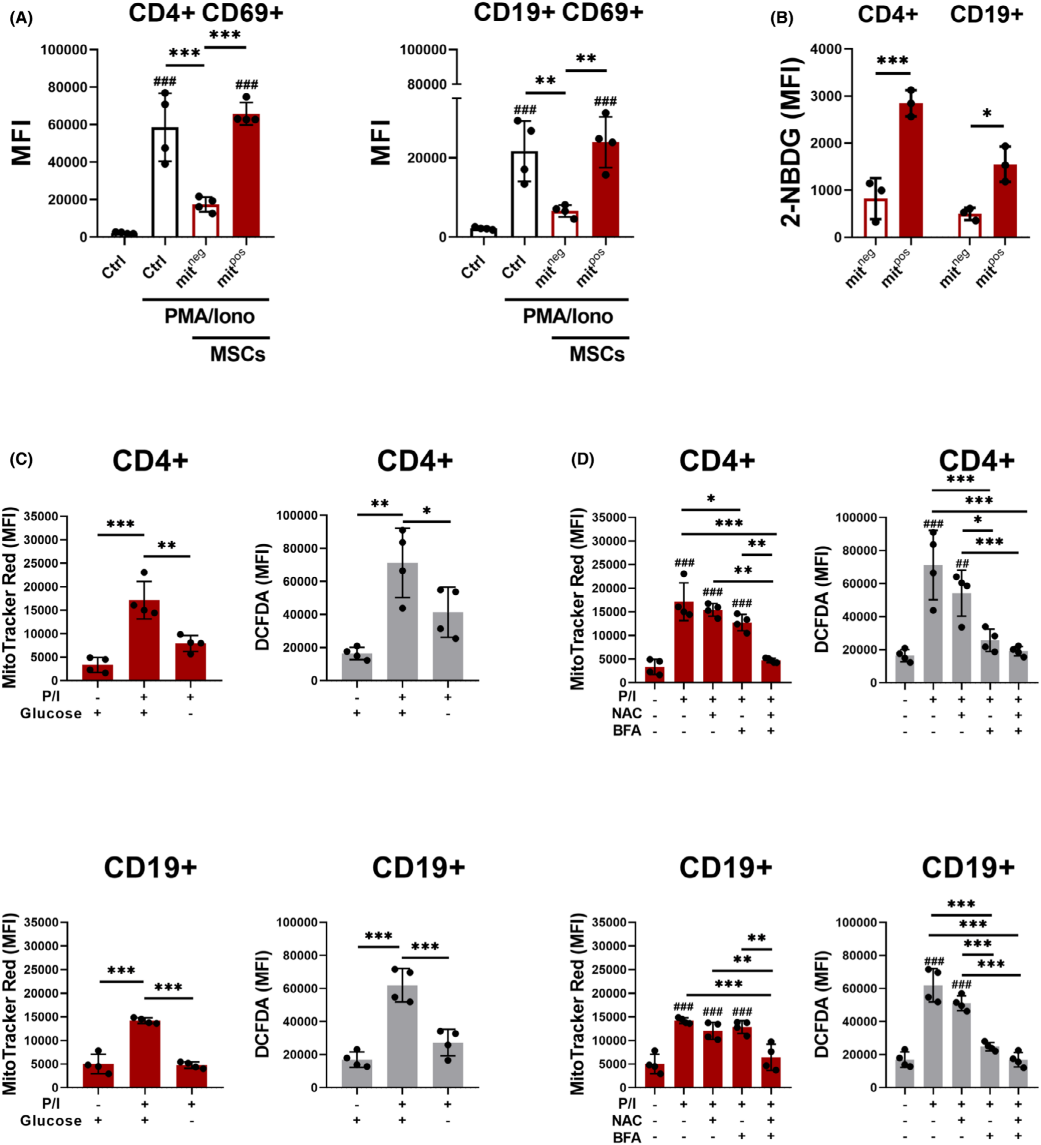
Factors and signals regulating MSC‐mediated mitochondrial transfer to CD4^+^ and CD19^+^ cells. (A) Mean fluorescence intensity (MFI) of the activation marker CD69 in cells that received mitochondria (mit^pos^) or did not receive mitochondria (mit^neg^) when co‐cultured with MitoTracker Red stained MSCs. Ctrl—control immune cells cultured without MSCs. PMA/Iono—immune cells pre‐treated with PMA and ionomycin for 24 h. MSCs—immune cells co‐cultured with MSCs. (B) Glucose uptake shown as MFI of fluorescently labelled glucose 2‐NBDG (.1 mg/mL) in mit^pos^ and mit^neg^ cells co‐cultured with MitoTracker Red‐stained MSCs. (C) Effect of the absence of glucose in the culture medium on the mitochondrial transfer from MSCs to activated CD4^+^ and CD19^+^ cells. Mitochondrial transfer is shown as MFI of MitoTracker Red and corresponding ROS production by these cells is shown as MFI of ROS marker DCFDA. (D) The effect of PMA/ionomycin (20 ng/mL), antioxidant NAC (5 mM) and protein trafficking inhibitor brefeldin A (BFA; 5 μg/mL) on the mitochondrial transfer from MSCs to CD4^+^ and CD19^+^ cells shown as MFI of MitoTracker Red and corresponding ROS production by these cells shown as MFI of DCFDA. All data are presented as mean ± SD of three–four independent experiments (*n* = 3–4), where *n* corresponds to the number of mice used. In all figures, statistically significant differences between groups are indicated by asterisks (*p < .05, **p < .01 and ***p < .001), hashtags show statistically significant differences between groups and Ctrl (^#^ *p* < .05, ^##^ *p <* .01 and ^###^ *p <* .001).

To gain deeper insight into the mechanism of mitochondrial transfer from MSCs to lymphocytes, we compared cells that acquired mitochondria with those that did not. In both CD4^+^ and CD19^+^ populations stimulated with PMA/ionomycin, mit^pos^ cells showed significantly higher expression of the activation marker CD69 (Figure 3A). The enhanced glycolytic activity, as another marker of activation, indicated by elevated glucose uptake in mit^pos^ cells, was confirmed using the 2‐NBDG assay (Figure 3B). The pre‐culturing of cells in glucose‐free medium for 24 h to restrict glycolysis and ensure optimal cell activation resulted in a reduction in mitochondrial transfer and ROS levels without affecting cell viability (Figure 3C), thus emphasising the importance of glycolysis in mitochondrial transfer.

Activation‐induced surface molecules may play a role in mitochondrial transfer during stimulation. To investigate this possibility, cells were treated with BFA, a disruptor of intracellular protein trafficking. While BFA or antioxidants alone did not result in a reduction of MitoTracker‐positive B cells, their combination led to a significant decrease in mitochondrial transfer. It is noteworthy that BFA alone reduced intracellular ROS levels, which suggests the presence of additional regulatory factors (Figure 3D).

### 2.4 Transfer of mitochondria from MSC to immune cells is bidirectional

**FIGURE 4:**
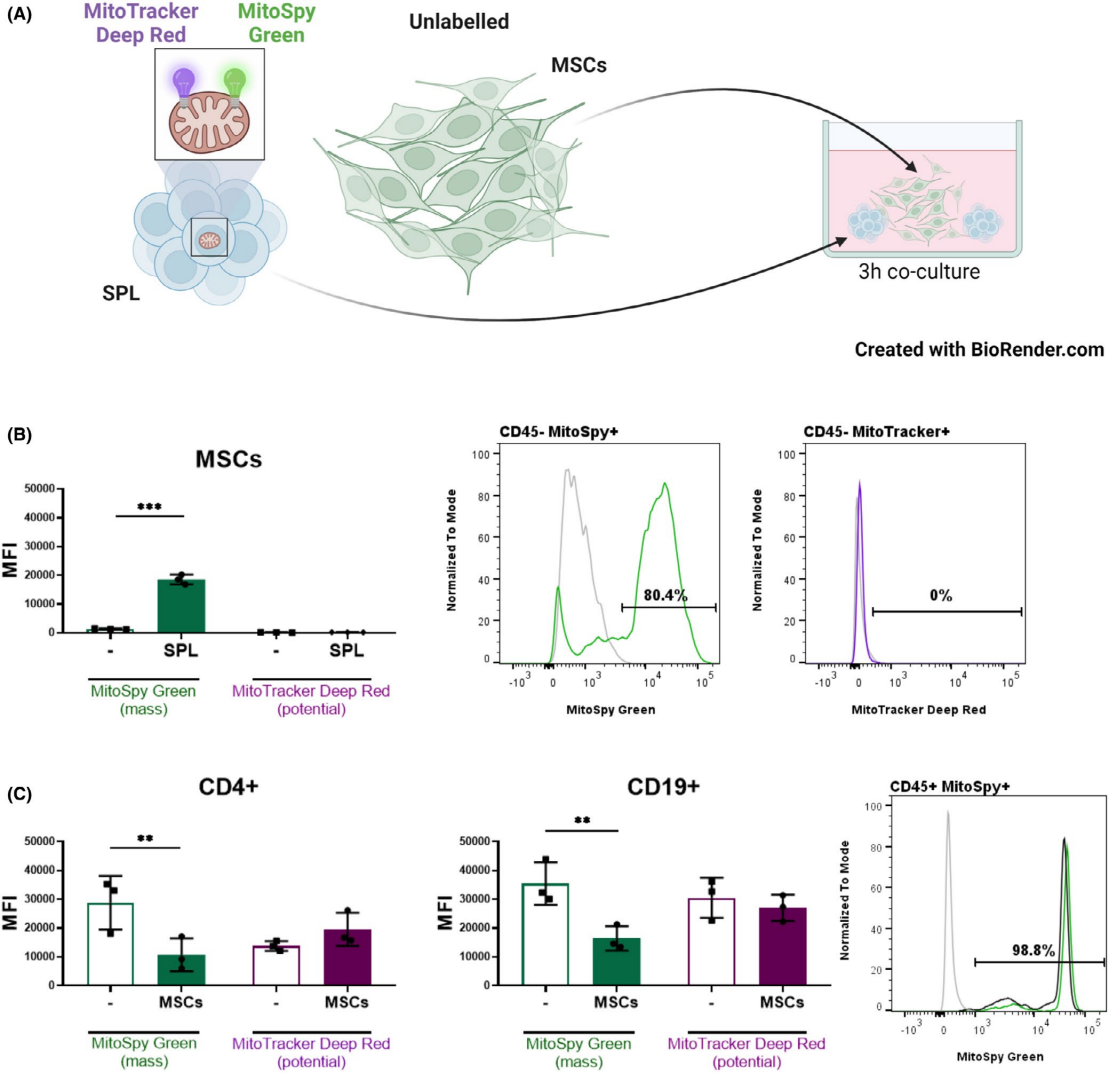
Bidirectional mitochondrial transfer. (A) Graphic depiction of the experiment. Splenocytes (SPL) were double‐stained with MitoTracker Deep Red and MitoSpy Green, then co‐cultured with unstained MSCs for 3 h. (B) MSCs received only mitochondria stained with MitoSpy Green (mitochondrial mass) but not with MitoTracker Deep Red (mitochondrial potential). The transfer of mitochondria from immune cells to MSCs is shown as the mean fluorescence intensity (MFI) of MitoSpy Green and MitoTracker Deep Red. Grey colour in histograms depict FMO control, green—MitoSpy Green, magenta—MitoTracker Deep Red. (C) Changes in mitochondrial composition in CD4^+^ and CD19^+^ immune cells without MSCs (−) or after the co‐culture with MSCs (MSCs) are shown as MFI of MitoSpy Green and MitoTracker Deep Red. Grey colour in histograms depict FMO control, green—MitoSpy Green, black—MitoSpy Green in splenocytes co‐cultured with MSCs. Data are expressed as mean ± SD of three independent experiments (*n* = 3), where *n* corresponds to the number of mice used. In all figures, statistically significant differences between groups are indicated by asterisks (*p < .05, **p < .01 and ***p < .001).

While these findings underscore the complex regulation of MSC‐mediated mitochondrial transfer to immune cells, we next explored whether this transfer is exclusively unidirectional. Specifically, we investigated whether mitochondria can also move from immune cells back to MSCs—a phenomenon that could shed light on mitochondrial dynamics and functionality in intercellular communication. The bidirectional nature of mitochondrial transfer is a topic of debate in the scientific community, but evidence suggests that it is possible.^6^, ^22^ The reduced frequency of B cells staining positive for MSC‐transferred mitochondria may indicate increased mitochondrial dynamics,^23^ potentially involving their return to donor cells. To explore this, splenocytes labelled with MitoTracker Deep Red and MitoSpy Green were co‐cultured with unlabeled MSCs (Figure 4A). MitoTracker Deep Red accumulation depends on mitochondrial membrane potential, while MitoSpy Green localizes to mitochondria regardless of membrane potential. Consequently, the combination of the two dyes can be used to determine whether mitochondria transferred from immune cells to MSCs are functional. Notably, only the green signal was detected within MSCs, which could indicate that only nonfunctional mitochondria are being transferred from spleen cells to MSCs (Figure 4B). This was supported by a decrease in MitoSpy Green intensity, but not MitoTracker Deep Red, in T and B cells after co‐culture (Figure 4C).

### 2.5 Mitochondrial transfer induces changes in genes associated with cell division in CD19 ^+^ cells

**FIGURE 5:**
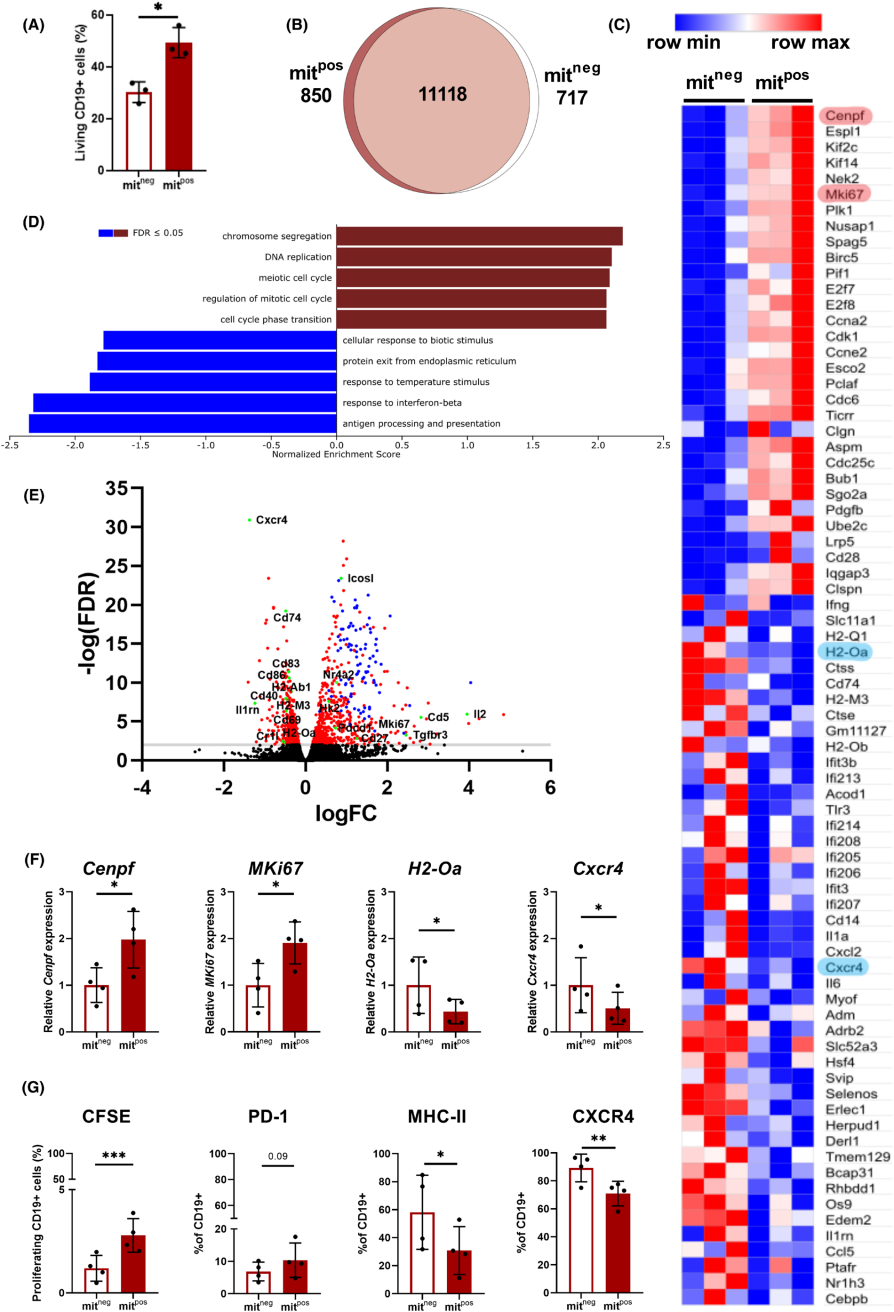
Bulk gene expression analysis of FACS‐sorted B cell acceptors and nonacceptors of mitochondria. (A) Survival rate cells that received mitochondria (mit^pos^) or did not receive mitochondria (mit^neg^) CD19^+^ cells cultured for 24 h after FACS‐sorting, shown as a percentage of living cells. (B) Venn diagram of differentially expressed (DE) genes from the whole transcriptome bulk RNA sequencing analysis of FACS‐sorted mit^neg^ and mit^pos^ CD19^+^ cells. (C) Heatmap of selected DE genes from the significantly enriched modules shown in (D). Normalized read counts were used as input. Highlighted genes were validated with qPCR as shown in (F). (D) Modules of significantly enriched genes (FDR ≤ .05) revealing pathways related to cell proliferation and antigen presentation in mit^pos^ in comparison to mit^neg^ CD19^+^ cells. Analysed with web‐based gene set enrichment analysis tool WebGestalt. DESeq2 calculated log2 fold changes were used as input. (E) Volcano plot of DE genes in mit^pos^ in comparison to mit^neg^ CD19^+^ cells showing some of the immunologically relevant DE genes (green dots) and DE genes associated with cell division and proliferation (blue dots). DESeq2 calculated –log(FDR) and log2 fold changes were used as input. (F) qPCR validation of some of the DE genes from bulk RNA sequencing analysis (highlighted in C). CD19^+^ mit^neg^ and mit^pos^ cells were FACS‐sorted and examined by real‐time qPCR. Data are presented as mean ± SD of four independent experiments. (G) FACS analysis of the marker of proliferation CFSE and protein levels of some of DE genes from bulk RNA sequencing analysis. CD19^+^ mit^neg^ and mit^pos^ cells were FACS‐sorted, stained with CFSE, and cultured for 24 h prior to FACS analysis. Data are presented as mean ± SD of four independent experiments (*n* = 4), where *n* corresponds to the number of mice used. In all figures, statistically significant differences between groups are indicated by asterisks (*p < .05, **p < .01 and ***p < .001).

Having established the bidirectional nature of mitochondrial transfer and its potential implications for mitochondrial dynamics, we next aimed to explore its functional relevance in immune cells, specifically in CD19+ B cells. For this purpose, splenocytes activated with PMA/ionomycin were co‐cultured with MitoTracker Red‐labelled MSCs to assess how MSC‐derived mitochondria influence gene expression and cellular behaviour. After a 3‐h co‐culture, we sorted CD19^+^ cells into mit^pos^ and mit^neg^ populations and then cultivated them for an additional 24 h. After this period, the sorted mit^pos^ cells exhibited higher viability compared to mit^neg^ cells, as indicated by the percentage of live cells (Figure 5A), along with cell count and WST assay results (Figure S5).

To further assess the impact of MSC‐derived mitochondria on B cell function, we employed bulk RNA‐seq (Figure S6) on activated, FACS‐sorted CD19^+^ mit^pos^ and mit^neg^ cells. Both groups were compared, and the obtained data were evaluated by gene set enrichment analysis. Our RNA‐seq data revealed that of 12685 total genes detected, 850 and 717 were differentially upregulated with statistical significance (FDR ≤ .05) in the mit^pos^ and mit^neg^ B cells, respectively (Figure 5B). Heat map and volcano plot show the differently expressed (DE) genes that were up‐ or down‐regulated in response to the acquisition of mitochondria (Figure 5C,E). Further, we used the web‐based gene set enrichment analysis tool WebGestalt to reveal overexpression of modules (FDR ≤ .05) containing genes related to cell division, including chromosome segregation, DNA replication and cell cycle. On the contrary, genes associated with antigen processing and presentation, as well as responsiveness to various exogenous stimuli, were decreased in mit^pos^ cells compared to mit^neg^ cells (Figure 5D).

Finally, we validated levels of selected immune‐response‐relevant DE genes involved in cell proliferation and antigen presentation by qRT‐PCR. The results confirmed significant changes in the expression of genes observed in the RNA‐Seq analyses of mit^pos^ compared with mit^neg^ cells (Figure 5F). Following validation of the transcript expression of selected genes, we aimed to confirm the corresponding protein changes in cells by flow cytometry. As shown by CFSE staining (Figure 5G), cell proliferation of mit^pos^ cells in culture was higher than that of mit^neg^ cells. Consistent with the qRT‐PCR and RNA‐seq results, significant changes in PD‐1, CXCR4 and MHCII proteins were observed in mit^pos^ and mit^neg^ cells (Figure 5G).

### 2.6 Transfer of mitochondria from MSCs reduces inflammation

**FIGURE 6:**
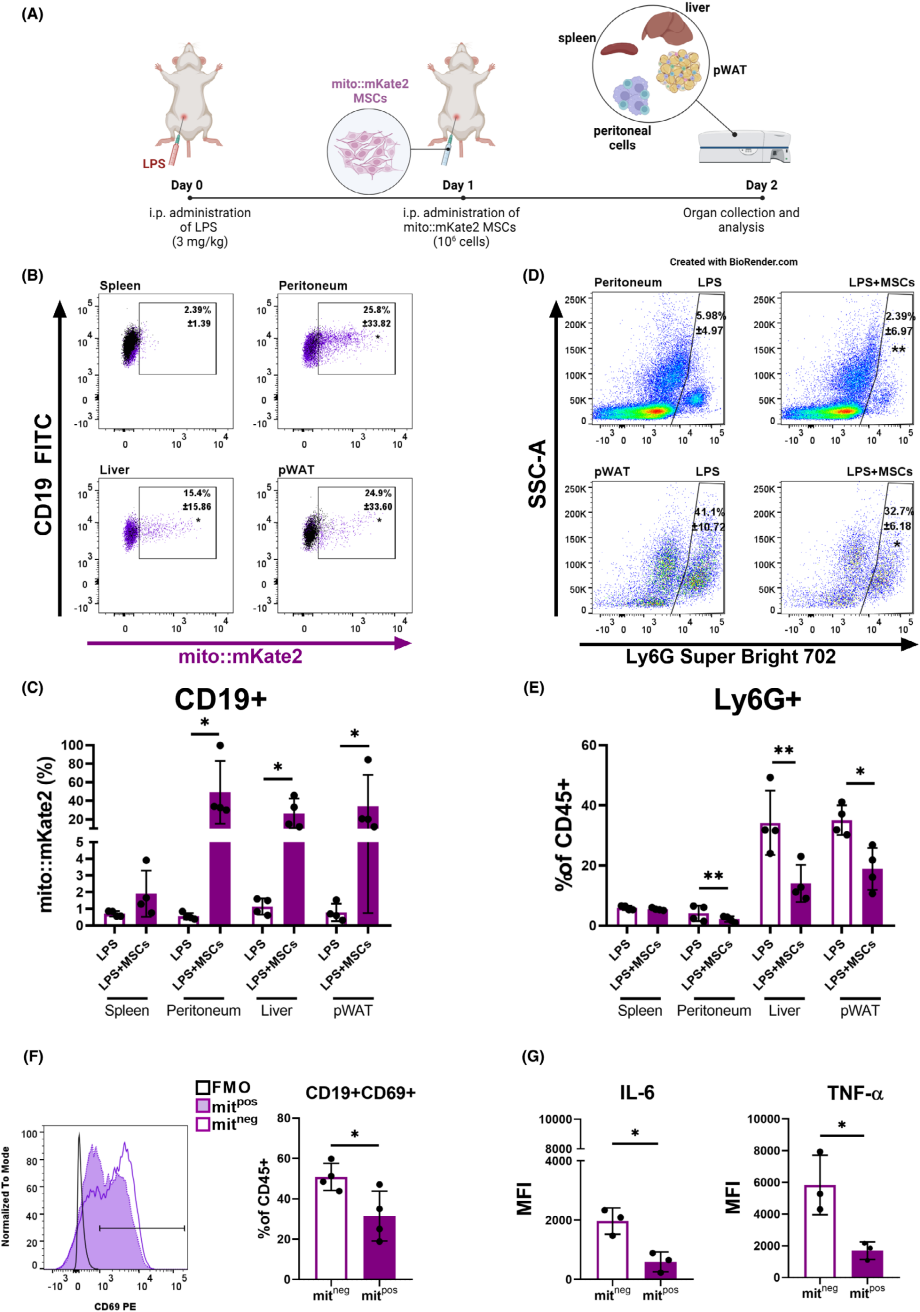
MSCs transfer mitochondria to CD19^+^ cells in vivo. MSCs isolated from mito::mKate2 transgenic mice were administered i.p. 24 h post LPS‐induced inflammation in BALB/c mice. Flow cytometry was employed to detect mitochondrial transfer and changes in immune cell populations. (A) Graphical depiction of the experimental design. BALB/c mice were injected i.p. with LPS on day 0 (LPS). On day 1 mito::mKate2 MSCs were administered i.p. (LPS + MSCs). Control mice (LPS) were injected with PBS. Mice were sacrificed on day 2. Organs including spleen, liver, visceral perigonadal WAT (pWAT) and peritoneal cells were collected and analysed with flow cytometry. (B) Representative dot plots and (C) graphs showing CD19^+^ cells positive for mito::mKate2 signal in peritoneum, liver, pWAT and spleen. (D) Representative dot plots and (E) graphs showing the LPS‐induced neutrophil (Ly6G^+^) infiltration and the effect of MSC administration. (F) Representative histogram and graph demonstrating the difference in CD69 expression between mit^neg^ and mit^pos^ CD19^+^ cells from peritoneal cavity. (G) Intracellular levels of pro‐inflammatory cytokines IL‐6 and TNF‐α in mit^neg^ and mit^pos^ CD19^+^ cells from peritoneal cavity. Data are presented as mean ± SD of three independent experiments (*n* = 3), where *n* corresponds to the number of mice used. In all figures, statistically significant differences between groups are indicated by asterisks (*p < .05, **p < .01 and ***p < .001).

Building on these findings, we next examined whether the functional changes induced by mitochondrial transfer to CD19+ cells in vitro could translate into immunomodulatory effects in vivo. To evaluate this, we utilized a model of LPS‐induced acute inflammation to investigate the capacity of MSCs for mitochondrial transfer and their potential role in reducing inflammation.^24^, ^25^ MSCs from mito::mKate2 mice were administered 24 h post‐LPS induction, and mitochondrial transfer to immune cells in the peritoneal cavity, WAT, liver and spleen was evaluated (Figure 6A). Approximately 30% of CD19^+^ cells in the peritoneal cavity, 20% in the liver and WAT, and fewer in the spleen exhibited mKate2 fluorescence (Figure 6B,C). Staining of MSCs derived from mito::mKate2 transgenic mice with MitoTracker Deep Red confirmed the transfer of functional mitochondria to CD19^+^ cells. The recruitment of neutrophils, which is linked to LPS‐induced inflammation, was reduced in the peritoneum, pWAT and liver, but not in the spleen (Figure 6D,E), indicating that MSC administration resulted in a reduction of inflammation. Remarkably, mit^pos^ CD19^+^ cells in the peritoneal cavity displayed lower CD69 expression, and reduced levels of IL‐6 and TNF‐α, indicating that acquiring mitochondria ultimately results in a less activated status of B cells (Figure 6F,G).

### 2.7 Artificial mitochondrial transfer reduces inflammation in B cells

**FIGURE 7:**
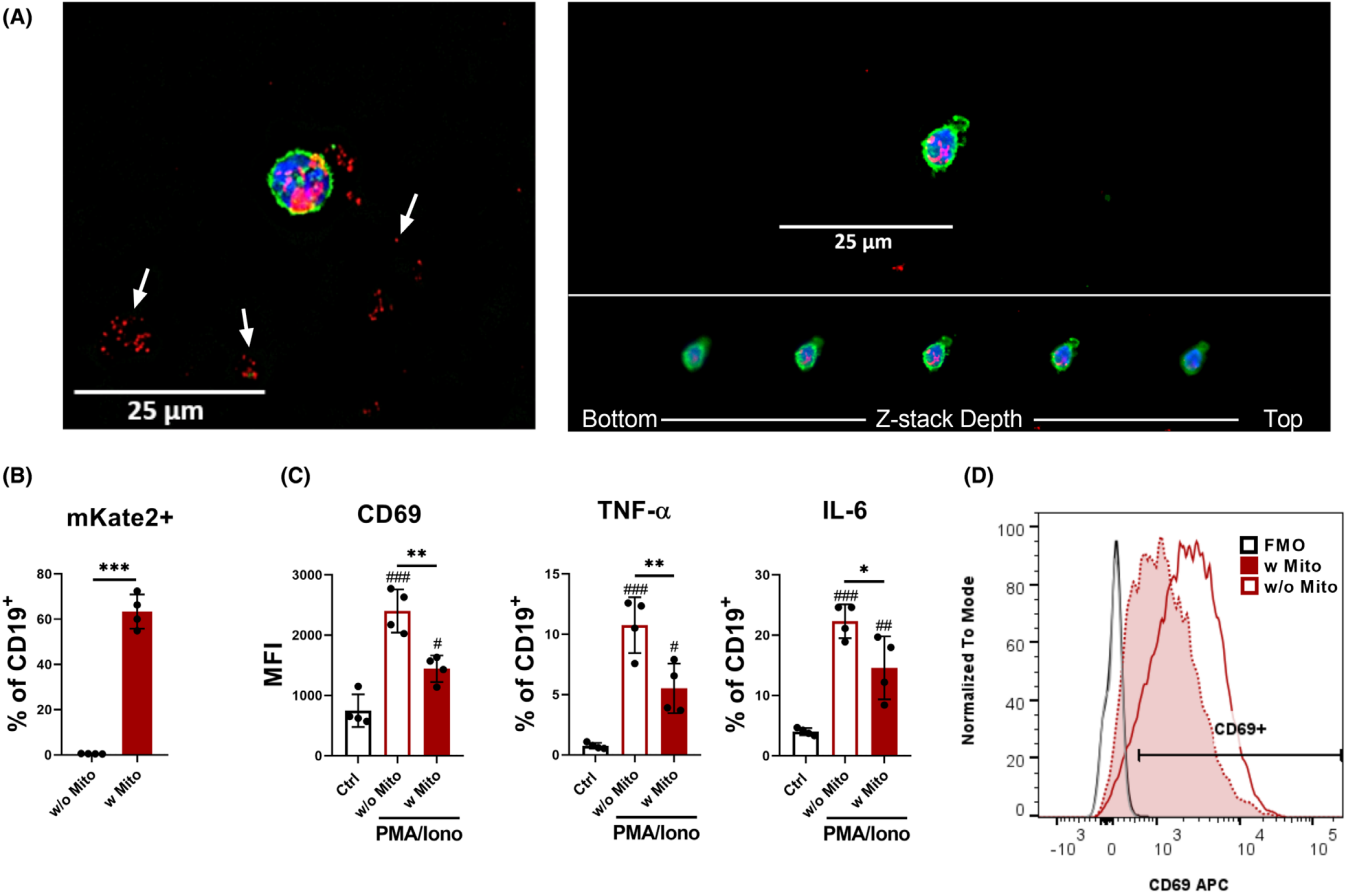
B cells incorporate mitochondria isolated from mito::mKate2 MSCs in a process of artificial mitochondrial transfer. (A) Isolated mitochondria in a co‐culture with FACS‐sorted CD19^+^ B cells. Mitochondria are visible both inside the cell and free in the culture (shown with arrows). The confirmation of the mitochondrial localization within the cell is illustrated by a series of z‐stacks captured from the bottom to the top. MKate2 mitochondria (red), Actin (green), nuclei stained with DAPI (blue). (B) Graph showing a percentage of mitochondria‐positive CD19^+^ cells after 48 h in culture. Cells were analysed with flow cytometry. (C) Graphs depicting the expression of surface molecules CD69 on CD19^+^ B cells (shown as MFI) and the percentage of TNF‐α and IL‐6‐producing CD19^+^ B cells. FACS‐sorted CD19^+^ B cells were cultured for 48 h unstimulated (Ctrl), or stimulated with PMA and Ionomycin without mitochondria (w/o Mito), or in the presence of mitochondria isolated from mito::mKate2 MSCs (w Mito). After this period, the cells were measured by flow cytometry. (D) Representative histogram demonstrating the difference in CD69 expression between Mito^−^ and Mito^+^ CD19^+^ B cells. Data are presented as mean ± SD of four independent experiments (*n* = 4), where *n* corresponds to the number of mice used. In all figures, statistically significant differences between groups are indicated by asterisks (*p < .05, **p < .01 and ***p < .001), hashtags show statistically significant differences between groups and Ctrl (^#^ *p* < .05, ^##^ *p <* .01 and ^###^ *p <* .001).

To confirm that the observed anti‐inflammatory phenotype in B cells was directly attributable to mitochondrial uptake, we conducted artificial mitochondrial transfer experiments using FACS‐sorted CD19^+^ B cells. These experiments allowed us to identify the specific effects of MSC‐derived mitochondria on B cell activation and cytokine production under controlled conditions. The immunosuppressive effect of isolated mitochondria has been demonstrated in T cells.^15^ Similar experiments were conducted to validate the role of MSC‐derived mitochondria in shaping the B cell phenotype. Specifically, B cells activated with PMA and ionomycin in the presence of mitochondria, isolated from the same number of MSCs used in our co‐culture system, showed decreased surface expression of the early activation marker CD69. Moreover, a reduced number of cells activated in the presence of mitochondria produced the pro‐inflammatory cytokines IL‐6 and TNF‐α (Figure 7C,D).

## 3 DISCUSSION

The transfer of mitochondria is emerging as one of the pivotal immunomodulatory mechanisms employed by MSCs, capable of reshaping immune cell function and inflammatory responses.^26^ While its effects on the phenotype of T‐cells^13^, ^14^, ^15^, ^16^, ^17^ and macrophages^10^, ^11^, ^12^ are known, the effect on B cells has not yet been described. This study addresses this gap by investigating how mitochondrial uptake influences the fate and functional properties of B lymphocytes. In agreement with previous research,^15^ we confirmed that MSCs transferred mitochondria to CD19^+^ cells with the least extent compared to other immune cell populations, an effect that we documented both in syngeneic^27^ and allogeneic settings. Interestingly, CD4^+^ and CD19^+^ lymphocytes benefited from mitochondrial uptake and improved survival. Although the underlying mechanisms are not fully understood, several possible mechanisms have been proposed to promote the survival of somatic or cancer cells after receiving mitochondria.^8^, ^28^, ^29^ These include increased ATP production, improved cellular bioenergetics, DNA repair, regulation of the Bax/Bcl‐2 balance, reduction of oxidative stress and increased resistance to cellular stress.

In the context of immune cell interactions, ROS‐induced mitochondrial transfer from MSCs to haematopoietic stem cells has been suggested to play a role in ‘emergency’ haematopoiesis. This transfer rapidly increased mitochondrial mass and induced metabolic changes, bypassing the delay associated with intracellular mitochondrial biogenesis.^30^ Inflammation, such as that induced by TNF‐α, has been suggested to trigger mitochondrial transfer, a process that is ROS‐dependent and inhibited by NAC.^31^ Our data suggest a possible role for ROS as a key factor, as we showed reduced mitochondrial transfer with antioxidants and a dose‐dependent correlation between ROS levels and mitochondrial transfer after cell activation.

Induction of mitophagy adds another layer of complexity to our findings, with at least 20% of cells exhibiting mitophagy exclusively after acquiring mitochondria from MSCs. Known for its pro‐survival role, mitophagy can enhance cell viability and direct immune cell differentiation through metabolic reprogramming.^27^ Notably, mitophagy promotes a metabolic switch toward glycolysis,^30^ which corresponds to our data showing increased glycolysis and activated phenotype of mit^pos^ CD4^+^ and CD19^+^ cells.

The question is whether the acquisition of mitochondria increases the release of ROS or whether increased ROS triggers mitochondrial transfer. Our study demonstrated that mitochondrial transfer is impaired in glucose‐free media, highlighting the importance of glycolysis and glucose, essential for immune cell activation.^32^ Interestingly, transfer occurred preferentially in activated cells. Neither antioxidants nor brefeldin A, which blocks intracellular protein transport and secretion, individually reduced mitochondrial transfer, despite lowering ROS levels and preventing EV release^33^ or extracellular marker expression.^34^ However, their combination effectively blocked mitochondrial transfer into CD19^+^ cells, suggesting that mitochondrial transport from MSCs to immune cells is a complex process involving factors beyond ROS and protein trafficking.

Several studies have suggested bidirectional intercellular mitochondrial transfer between MSCs and somatic or cancer cells, impacting both donor and recipient cells.^1^, ^8^, ^22^, ^31^, ^35^ This process offers a potential mechanism for replacing dysfunctional mitochondria with functional counterparts, thus restoring aerobic respiration and effectively repairing damaged tissues. Our study demonstrates, for the first time, that immune cells transfer dysfunctional mitochondria to MSCs and receive functional ones in return. This exchange induces metabolic reprogramming in immune cells, shifting them toward oxidative phosphorylation, enhancing survival and supporting energy‐intensive immune responses. Notably, this process favours an anti‐inflammatory phenotype.^36^

Consistent with studies documenting the beneficial, pro‐survival effects of MSC‐mediated mitochondrial transfer in both animal models and humans,^10^, ^12^, ^37^, ^38^ our results showed that CD19^+^ cells pre‐treated with a combination of PMA and ionomycin and subsequently receiving mitochondria exhibited higher viability and metabolic activity compared to their counterparts that did not receive mitochondria. These cells upregulated genes associated with cell division and downregulated those associated with antigen presentation. These findings align with previous reports documenting mitochondrial transfer as a pro‐survival mechanism. In addition, gene and protein expression data suggest that these cells adopt an anti‐inflammatory phenotype following mitochondrial uptake.

In vivo, mitochondrial transfer from MSCs to various cell types has been documented to rescue tissue damage.^10^, ^11^, ^39^, ^40^ While macrophages have been specifically identified as recipients of mitochondria in vivo,^10^, ^11^ in vitro evidence shows that different cell types can acquire mitochondria,^15^ leading to the speculation that this phenomenon may extend to in vivo scenarios. Our study provides evidence that B cells can, indeed, acquire functional mitochondria from MSCs in vivo. This transfer may involve active mechanisms or engulfment of apoptotic MSCs by phagocytes. Although our experimental setup does not allow definitive determination of the exact mechanism, the transfer of functional mitochondria supports an active MSC‐driven process.

Several studies have highlighted the anti‐inflammatory effects of MSC administration in attenuating LPS‐induced inflammation as evidenced by reduced neutrophil infiltration,^41^ lower levels of proinflammatory cytokines, including IL‐6 and TNF‐α,^25^, ^41^ attenuated cell apoptosis^25^ and amelioration of tissue damage.^25^, ^42^ As the acquisition of mitochondria negatively correlated with the level of activation and production of inflammatory cytokines in B cells, this study further highlights the involvement of mitochondrial trafficking in the anti‐inflammatory effects of MSCs.

While the immunomodulatory effects of mitochondrial transplantation using isolated mitochondria are well‐documented^15^, ^43^ their impact on B cells has been less explored. Here, we provide evidence that mitochondria isolated from MSCs, when transferred to purified CD19^+^ B cells, exhibit anti‐inflammatory effects similar to those observed in our mixed in vitro and in vivo models.

In conclusion, this study demonstrates that MSCs transfer mitochondria to CD19^+^ cells, although less efficiently than to other immune populations. Mitochondrial transfer was influenced by ROS levels, with a bidirectional exchange observed—immune cells transferred dysfunctional mitochondria to MSCs and received functional mitochondria. CD19^+^ cells receiving mitochondria showed increased viability, metabolic activity and altered gene expression related to cell division and antigen presentation. The LPS‐induced acute inflammation model confirmed in vivo mitochondrial transfer to B cells across organs. These findings extend the understanding of the complex relationship between MSCs and immune cells by means of mitochondrial trafficking and provide insights into potential therapeutic applications for modulation of immune cell function.

## 4 MATERIALS AND METHODS

### 4.1 Animals

BALB/c mice of both genders were purchased from AnLab s.r.o., Prague, Czech Republic. Mito::mKate2 transgenic mice (C57BL/6J background) ubiquitously express mitochondria‐localized far‐red mKate2 fluorescent protein.^44^ Expression of this protein is heterozygous. Genotyping and verification of fluorescence were done on mouse tails using fluorescence microscope Leica DMIL LED. Transgenic mice were bred in‐house. All animals were housed in a specific pathogen‐free facility and used for experiments at 7–12 weeks of age.

### 4.2 Cell isolation

Adipose tissue‐derived MSCs were isolated from inguinal fat pads of the BALB/c or mito::mKate2 mice as was described previously.^45^, ^46^ Harvested MSCs were then cultured in high‐glucose Dulbecco's modified Eagle medium (DMEM; Merck, St. Louis, MO, USA) supplemented with 10% FBS (Biosera, Cholet, France), antibiotics (100 mg/mL of streptomycin, 100 U/mL of penicillin) and 10 mM Hepes buffer. Passages 3–5 were used for experiments.

Splenocytes were isolated by mechanical homogenization of the spleen of the BALB/c mice. All experiments involving splenocyte culture or co‐culture were conducted using RPMI 1640 medium (Merck) supplemented with 10% FBS (Merck), antibiotics (100 mg/mL of streptomycin, 100 U/mL of penicillin) and 10 mM Hepes buffer.

### 4.3 Cell culture conditions

Splenocytes (1.5 × 10^7^) were cultured in 2 mL of RPMI 1640 medium in a 6‐well plate. The cells were either left unstimulated or stimulated with 5 mM N‐acetylcysteine (NAC; Merck), 5 μM hydrogen peroxide (H_2_O_2_; Merck), or activated with either lipopolysaccharide (LPS; Merck) at 1.25 μg/mL or phorbol 12‐myristate 13‐acetate (PMA; Merck) at 1, 5, 10 or 20 ng/mL and ionomycin (Merck) at 1 μg/mL for 24 h. Alternatively, splenocytes were activated for 24 h with PMA/Ionomycin at 20 ng/mL in the presence of 5 mM NAC, Brefeldin A (Thermo Fisher Scientific, Waltham, MA, USA) at 5 μg/mL, or in 10% RPMI 1640 medium without glucose. The splenocytes were then directly analysed or co‐cultured with MSCs.

### 4.4 Staining of mitochondria

MSCs were stained with MitoTracker Red CMXRos (Thermo Fisher), which marks functional mitochondria based on their membrane potential. The staining procedure was performed using a 100 nM solution of MitoTracker for 30 min at 37°C. This concentration was chosen as it is nontoxic for cells. No dye leakage was observed from the stained cells. After the cultivation period, the cells were rinsed with warm phosphate buffer (PBS) and collected for subsequent manipulation.

### 4.5 Mitochondria isolation

Mitochondria were isolated from the MSCs following a previously established protocol as described.^47^ Briefly, MSCs were resuspended in the isolation buffer containing 250 mM sucrose, 10 mM Tris/HCl and .5 g/L of fatty acid‐free BSA, pH 7.2, and homogenized by passing them through a narrow 30G insulin needle (Omnican). To prevent mitochondrial damage, the cells were maintained on ice throughout the process. The homogenate was subjected to differential centrifugation. First, the sample was centrifuged at 600 × *g* for 10 min at 4°C to remove cellular debris and nuclei. The resulting supernatant, enriched with mitochondria, was transferred to fresh Eppendorf tubes and centrifuged at 8000 × *g* for 10 min at 4°C to pellet the mitochondria. The mitochondrial pellet was gently resuspended in an appropriate culture medium. To facilitate artificial mitochondrial transfer, the mitochondrial suspension was added directly to the recipient cell culture, and the culture plates were centrifuged at 600 × *g* for 4 min.

### 4.6 Co‐culture conditions

MSCs stained with MitoTracker Red CMXRos (5 × 10^4^) were co‐cultured with either unstained splenocytes or splenocytes stained with MitoSpy Green or MitoTracker Deep Red (10^6^) in a 24‐well plate at a 1:20 ratio for 3 h. Alternatively, cells were co‐cultured in a Transwell system where donor and acceptor cells were separated by a membrane with 3 μm pores (Corning Transwell Multiple Well Plate with Permeable Polyester Membrane Inserts; Merck). The cells were then harvested for further analysis.

### 4.7 Flow cytometry

The harvested cells were washed with PBS/.5% bovine serum albumin (BSA) and incubated for 30 min at 4°C with conjugated monoclonal antibodies (see Table 1). Dead cells were determined with Hoechst 33258 (Merck) added 10–15 min before the assay (see Gating Strategy in S1). To detect intracellular cytokines, fixable viability dye LIVE/DEAD Fixable Violet (Thermo Fisher) was used for dead cell determination. Cells were then fixed and permeabilized with Fixation and Permeabilization Kit (Thermo Fisher) according to the manufacturer's protocol. Intracellular staining was then performed using conjugated monoclonal antibodies (see Table 1). Further analysis was performed using an LSR II (BD Bioscience) flow cytometer and subsequent gating using FlowJo software (FlowJo BD, Ashland, OR, USA) (see Gating Strategy in S2).

**TABLE 1:** List of conjugated antibodies used in flow cytometric analysis.

Target	Fluorochrome	Clone	Company	CAT	Dilution
CD45	AF700	30‐F11	BioLegend	103128	1:150
CD4	FITC	GK1.5	BioLegend	100406	1:160
CD4	APC	GK1.5	BioLegend	100412	1:160
CD4	PE	GK1.5	BioLegend	100408	1:160
CD8a	FITC	53–6.7	BioLegend	100706	1:160
CD8a	APC	53–6.7	BioLegend	100712	1:160
CD19	FITC	6D5	BioLegend	115506	1:90
CD19	APC	6D5	BioLegend	115512	1:90
CD19	PE	6D5	BioLegend	115508	1:90
CD11b	FITC	M1/70	BioLegend	101206	1:100
CD11b	APC	M1/70	BioLegend	101212	1:100
MHC II	FITC	M5/114.15.2	BioLegend	107606	1:75
Ly6G	SB702	1A8‐Ly6G	Invitrogen	67‐9668‐82	1:100
PD‐1	FITC	29F.1A12	BioLegend	135214	1:65
CXCR4	APC	L276F12	BioLegend	146508	1:100
IL‐6	APC	MP5‐20F3	BioLegend	504508	1:80
TNF‐α	PE	TN3‐19.12	Invitrogen	506104	1:100

FACS sorting of acceptors and nonacceptors of mitochondria was performed after surface marker staining on FACS Aria IIu. Cells were harvested and stained in HBSS (without Ca^2+^/Mg^2+^) with 1% BSA and .02 mg/mL DNase I. Propidium Iodide was used to determine dead cells. The sorted cells were collected into FBS for further culture or for RNA isolation.

### 4.8 Fluorescence microscopy

MSCs were co‐cultured with splenocytes at a 1:20 ratio for at least 3 h to allow their adhesion, washed with PBS/.5% BSA, and fixed using 4% paraformaldehyde in PBS for 10 min. Samples were then permeabilized and blocked with .2% Triton x‐100 and 1% bovine serum albumin (BSA) in PBS, and stained for 1 h with rat anti‐mouse CD45 primary antibody (1:200; Biolegend) and mouse anti‐mouse α‐tubulin primary antibody (1:200; Invitrogen), and subsequently for 1 h with Alexa Fluor 647‐labelled goat anti‐rat antibody (1:400; Invitrogen) or Alexa Fluor 488‐labelled goat anti‐mouse antibody (1:400; Invitrogen). Alternatively, to α‐tubulin, an Alexa Fluor 488 Phalloidin probe was used to stain actin in cells. Nuclei were stained with DAPI in the mounting Mowiol solution (Merck). Mitochondrial transfer from MSCs to CD45^+^ cells was then observed using the Leica Dmi8 fluorescent microscope (Leica Microsystems, Wetzlar, Germany).

### 4.9 Reactive oxygen species production

2′,7′‐Dichlorofluorescin diacetate 15 μM (DCFDA, Merck) was used to detect ROS production. The staining was performed for 30 min at 37°C according to the manufacturer's instructions; the cells were then washed with PBS/.5% BSA, stained for surface markers and assessed using flow cytometry.

### 4.10 Glucose uptake measurement

Splenocytes were activated with PMA/Ionomycin for 24 h and then co‐cultured with MitoTracker CMXRos Red‐stained MSCs for 3 h in 10% RPMI 1640 medium without glucose. The fluorescent analogy of glucose 2‐NBDG (2‐(N‐(7‐nitrobenz‐2‐oxa‐1,3‐diazol‐4‐yl)amino)‐2‐deoxyglucose) was added to the co‐culture system at .1 mg/mL. After 20 min‐long incubation at 37°C, cells were washed with PBS/.5% BSA, stained for surface markers, and assessed using flow cytometry.

### 4.11 Proliferation

A proliferation assay was performed with FACS‐sorted CD19^+^ cells using a 5 μM CFSE staining solution. After 20 min incubation at RT, cells were washed twice with warm 10% RPMI 1640 medium and cultured for 24 h. Harvested cells were further analysed using flow cytometry.

### 4.12 Apoptosis

To evaluate the survival rate of splenocytes, the cells were stained for surface markers, washed with PBS/.5% BSA, and then stained with the Annexin V detection kit for 15 min at room temperature, following the manufacturer's protocol (EXBIO, Prague, Czech Republic). Dead cells were identified using Hoechst 33258 and measured using flow cytometry.

### 4.13 Mitophagy

To detect mitophagy, we performed staining using a Mitophagy detection kit following the manufacturer's protocol (Dojindo, Munich, Germany). The staining was carried out for 30 min at 37°C. After staining, cells were washed twice with warm RPMI 1640 medium without serum and then with PBS/.5% BSA. Finally, the cells were stained for surface markers and assessed using flow cytometry.

### 4.14 WST assay

The WST‐1 assay (Roche, Basel, Switzerland) was used to determine the activity of mitochondrial succinate dehydrogenase. After 3 h of co‐culture of splenocytes with MSCs, CD19^+^ cells were sorted based on the positivity for the MitoTracker dye. Subsequently, mitochondria recipient or nonrecipient cells were incubated at an initial density of 2 × 10^5^ cells per well in a 96‐well plate for 24 h. The WST‐1 assay was performed according to the manufacturer's protocol.

### 4.15 qRT‐PCR

Mitochondrial recipient and nonrecipient CD19^+^ cells were FACS‐sorted after co‐culture with donor MSCs. RNA was then isolated from these cells using TRIreagent® (Molecular Research Center, Cincinnati, OH, USA) according to the manufacturer's protocol. Isolated RNA was used for reverse transcription using SuperScript™ IV Reverse Transcriptase (Thermo Fisher), incorporating RNaseOUT™ Recombinant Ribonuclease Inhibitor (Thermo Fisher) and Random Hexamer Primer (Thermo Fisher) according to the specified instructions. Quantitative PCR was then performed using HOT FIREPol® EvaGreen® qPCR Mix Plus (Solis BioDyne, Tartu, Estonia) following the manufacturer's protocol and was assessed using LightCycler480 (Roche). *Gapdh* and *Actb* were used as housekeeping genes. The list of primers is in the Supplementary Material (see Table 2).

**TABLE 2:** Sequences of primers used in real‐time qPCR.

Primers	Official Full Name	NCBI Gene	Sequence
*Actb*	Actin b	11461	ATGGAGCCACCGATCCACA
			CATCCGTAAAGACCTCTATGCCAAC
*Gapdh*	Glyceraldehyde‐3‐phosphate dehydrogenase	14433	GCCAAGGTCATCCATGACAAC
			GTCCACCACCCTGTTGCTGTA
*Mki67*	Antigen identified by monoclonal antibody Ki 67	17345	ATCATTGACCGCTCCTTTAGGT
			GCTCGCCTTGATGGTTCCT
*Cenpf*	Centromere protein F	108000	GCTCAGCTTTTGCACCAGG
			AGGCGTAGTTCTAACTCAGTCAT
*Cxcr4*	C‐X‐C motif chemokine receptor 4	12767	GACTGGCATAGTCGGCAATG
			AGAAGGGGAGTGTGATGACAAA
*H2‐Oa*	Histocompatibility 2, O region alpha locus	15001	TCTACCAATCTTACGACGCTTCT
			CACACGACCTCCTCGTTCT

### 4.16 Bulk RNA‐sequencing

B cells were stained with Alexa Fluor 700‐labelled mAb CD45 and APC‐labelled mAb CD19 in HBSS (without Ca^2+^/Mg^2+^) with 1% BSA and .02 mg/mL DNAse for 30 min. FACS sorting was performed in the same solution to prevent cell clumping. Mitochondrial acceptor and nonacceptor B cells were sorted and then added into the lysis buffer of an E.Z.N.A.® Microelute Total RNA kit (Omega Bio‐tek, Norcross, GA, USA). RNA was isolated following cell lysis and homogenization according to the manufacturer's protocol. Isolated RNA was used for library preparation by means of the DNBSEQ Eukaryotic Strand‐specific mRNA library kit/protocol. Libraries were then sequenced on the BGI DNBseq platform in the PE100 regime. The reference GRCm39 mouse genome and annotation were downloaded from RefSeq (2023‐05‐29, https://www.ncbi.nlm.nih.gov/refseq/). Read quality was checked using FastQC version 0.11.9 (https://www.bioinformatics.babraham.ac.uk/projects/fastqc/). Adapters and low‐quality sequences were removed using Trimmomatic .39 (PMID: 24695404). Clean reads were aligned to the mouse genome using HISAT2 2.2.1^48^ and SAMtools 1.13.^49^, ^50^ Read coverage tracks were then computed and normalized to the respective mapped library sizes using deepTools 3.5.1 (PMID: 27079975). Mapped reads and coverage data were inspected visually in the IGV 2.9 browser (PMID: 21221095). Differentially expressed genes were detected using the DESeq2 packages (PMID: 25516281) in R/Bioconductor (PMID: 15461798; PMID: 25633503). Gene‐set enrichment analysis was performed using WEB‐based Gene SeT AnaLysis Toolkit (WebGestalt) (PMID: 31114916). Heatmaps were created using the Phantasus application.^51^

### 4.17 Model of LPS‐induced inflammation

Female BALB/c mice (7–12 weeks old) were injected intraperitoneally (i.p.) with LPS at 3 mg/kg to induce inflammation. After 24 h, MSCs isolated from mito::mKate2 mice were injected i.p. into the mice in the LPS + MSCs group at 10^6^ cells in 200 μL of sterile PBS. The control group of only LPS‐injected mice (LPS) was subjected to i.p. injection of PBS. Each group consisted of at least 3 animals (see Figure 6A for experimental design). Mice were sacrificed 48 h after LPS administration. Cells isolated from the peritoneum, peritoneal white adipose tissue (WAT), spleen and liver were further assessed using flow cytometry.

### 4.18 Statistical analysis

Statistical analysis was performed using The Prism software (GraphPad Software, San Diego, CA, USA). Data are shown as mean ± standard deviation (SD) of at least three independent experiments. Statistical significance among individual groups was determined by means of one‐way analysis of variance (ANOVA) along with the Tukey or Dunnett post hoc test or Student's t‐test in the case of a two‐group comparison. Differences with *p* < .05 were considered statistically significant.

## AUTHOR CONTRIBUTIONS

VS and MK designed the study. VS, BP, NF, DV and NJ performed experiments. VS, MP and MK analyzed data. VS and MK prepared the manuscript. MP, ZN and JN critically revised the article. ZN and JN provided transgenic animals. VS, MK and JN provided funding acquisition. All authors read and approved the final manuscript.

## CONFLICT OF INTEREST STATEMENT

The authors declare no competing interests.

## Supporting information


**Figure S1:** Gating strategy of mitochondrial transfer to CD45^+^CD19^+^ or CD45^+^Ly6G^+^ immune cells. Gating strategy: debris was excluded based on forward and side scatter, then doublets were excluded, and immune cells were gated based on CD45 positivity on Hoechst 33258 negative population (live). (A) Splenocytes isolated from BALB/c mice were analysed to determine mitochondrial transfer after 3 h co‐culture with MSCs labelled with MitoTracker Red CMXRos by flow cytometry. From CD45^+^ cells, B cells were gated as CD19^+^ cells. MitoTracker Red positivity on CD19^+^ cells (CD19^+^MTR^+^) was determined based on the FMO control. (B) Peritoneal cells isolated from BALB/c mice were analysed to determine Ly6G^+^ neutrophile, granulocyte and monocyte infiltration.
**Figure S2:** Gating strategy of TNF‐α stained as intracellular protein. Debris was excluded based on forward and side scatter, then doublets were excluded, and immune cells were gated based on CD45 positivity on LIVE/DEAD Fixable violet negative population (live). From CD45^+^ cells, B cells were gated as CD19^+^ cells. Acceptors and nonacceptors of mitochondria were gated as mKate^+^ and mKate^−^ cells. From both these populations TNF‐α positive cells were determined.
**Figure S3:** Mitochondrial transfer in a contactless co‐culture system. MitoTracker Red‐stained MSCs were co‐cultured with immune cells in a double chamber TransWell system. Cells were separated by a membrane with 3 μm pores. Ctrl – cells co‐cultured in a standard single chamber system. TW – cells co‐cultured in a double chamber TransWell system. Data are presented as mean ± SD of three independent experiments (*n* = 3), where *n* corresponds to the number of mice used. Statistically significant differences between groups are indicated by asterisks (*p < .05, **p < .01 and ***p < .001).
**Figure S4:** The effect of ROS production and mitophagy on mitochondrial transfer. (A) ROS production by different immune cell populations, shown as the mean fluorescence intensity (MFI) of the ROS marker DCFDA. Data are presented as mean ± SD of four independent experiments. (B) MSC‐derived mitochondrial transfer and corresponding ROS production by CD19^+^ immune cells, shown as a percentage of MitoTracker Red positive cells or MFI of ROS marker DCFDA. Prior to co‐culture with MSCs, immune cells were cultured for 24 h either untreated or in the presence of 5 μM hydrogen peroxide, 5 mM NAC, or a combination of both. Data are presented as mean ± SD of four independent experiments (*n* = 4), where *n* corresponds to the number of mice used. (C) Mitophagy in CD19^+^ immune cells which were cultured as mentioned previously, shown as a percentage of MitophagyDye positive cells. Data are presented as mean ± SD of three independent experiments (*n* = 3), where *n* corresponds to the number of mice used. In all figures, statistically significant differences between groups are indicated by asterisks (*p < .05, **p < .01 and ***p < .001).
**Figure S5:** The effect of mitochondrial transfer on CD19^+^ cells. (A) Survival rate cells that received mitochondria (mit^pos^) or did not receive mitochondria (mit^neg^) CD19^+^ cells cultured for 24 h after FACS‐sorting, shown as counts of living cells. (B) Analysis of metabolic activity of mit^neg^ and mit^pos^ CD19^+^ cells using the WST‐1 assay 24 h after FACS‐sorting. Data are presented as mean ± SD of three independent experiments (*n* = 3), where *n* corresponds to the number of mice used. Statistically significant differences between groups are indicated by asterisks (*p < .05, **p < .01 and ***p < .001).
**Figure S6:** Principal component analysis (PCA) plot of normalized gene counts. Principal component (PC) 1 (red) versus PC2 (blue) coloured by means of mitochondria acquirement. Mit^neg^ and mit^pos^ samples coming from the same mouse are connected with dotted lines.
**Figure S7:** Protective and pro‐survival effect of mitochondrial transfer. (A) Relative expression of genes *Tfam* and *Polg*, which are associated with mtDNA protection and transcription, as well as apoptosis‐related genes *Bcl2* and *Bax* obtained from the RNA‐sequencing data. (B) Relative expression of genes *Bcl2* and *Bax* obtained from qPCR analysis. Data are presented as mean ± SD of three independent experiments. (*n* = 3), where n corresponds to the number of mice used. Statistically significant differences between groups are indicated by asterisks (*p < .05, **p < .01 and ***p < .001).

## Data Availability

All data supporting the findings of this study are available within the article and the supplemental information, or from the corresponding author upon reasonable request. All scripts used for RNA‐seq data analysis are available at https://github.com/mprevorovsky/krulova‐mice‐mitochondria. The raw and processed RNA‐seq data are available from the ArrayExpress database (https://www.ebi.ac.uk/biostudies/arrayexpress) under the accession number E‐MTAB‐13438.

## References

[1] Dong LF , Rohlena J , Zobalova R , et al. Mitochondria on the move: horizontal mitochondrial transfer in disease and health. J Cell Biol. 2023;222(3):e202211044. doi:10.1083/JCB.202211044/213873 36795453 PMC9960264

[2] Yan W , Diao S , Fan Z . The role and mechanism of mitochondrial functions and energy metabolism in the function regulation of the mesenchymal stem cells. Stem Cell Res Ther. 2021;12(1):140. doi:10.1186/s13287-021-02194-z 33597020 PMC7890860

[3] Huang T , Zhang T , Gao J . Targeted mitochondrial delivery: a therapeutic new era for disease treatment. J Control Release. 2022;343:89‐106. doi:10.1016/J.JCONREL.2022.01.025 35077740

[4] Paliwal S , Chaudhuri R , Agrawal A , Mohanty S . Human tissue‐specific MSCs demonstrate differential mitochondria transfer abilities that may determine their regenerative abilities. Stem Cell Res Ther. 2018;9(1):1‐9. doi:10.1186/S13287-018-1012-0 30409230 PMC6225697

[5] Lee H , Jeong OY , Park HJ , et al. Promising therapeutic effects of embryonic stem cells‐origin mesenchymal stem cells in experimental pulmonary fibrosis models: immunomodulatory and anti‐apoptotic mechanisms. Immune Netw. 2023;23(6):e45. doi:10.4110/IN.2023.23.E45 38188598 PMC10767550

[6] Liu Z , Sun Y , Qi Z , Cao L , Ding S . Mitochondrial transfer/transplantation: an emerging therapeutic approach for multiple diseases. Cell Biosci. 2022;12(1):66. doi:10.1186/S13578-022-00805-7 35590379 PMC9121600

[7] Mukkala AN , Jerkic M , Khan Z , Szaszi K , Kapus A , Rotstein O . Therapeutic effects of mesenchymal stromal cells require mitochondrial transfer and quality control. Int J Mol Sci. 2023;24(21):15788. doi:10.3390/IJMS242115788 37958771 PMC10647450

[8] Liu D , Gao Y , Liu J , et al. Intercellular mitochondrial transfer as a means of tissue revitalization. Signal Transduct Target Ther. 2021;6(1):1‐18. doi:10.1038/s41392-020-00440-z 33589598 PMC7884415

[9] Zampieri LX , Silva‐Almeida C , Rondeau JD , Sonveaux P . Mitochondrial transfer in cancer: a comprehensive review. Int J Mol Sci. 2021;22(6):3245. doi:10.3390/IJMS22063245 33806730 PMC8004668

[10] Morrison TJ , Jackson M v , Cunningham EK , et al. Mesenchymal stromal cells modulate macrophages in clinically relevant lung injury models by extracellular vesicle mitochondrial transfer. Am J Respir Crit Care Med. 2017;196(10):1275‐1286. doi:10.1164/rccm.201701-0170OC 28598224 PMC5694830

[11] Jackson MV , Morrison TJ , Doherty DF , et al. Mitochondrial transfer via tunneling nanotubes is an important mechanism by which mesenchymal stem cells enhance macrophage phagocytosis in the in vitro and in vivo models of ARDS. Stem Cells. 2016;34(8):2210‐2223. doi:10.1002/STEM.2372 27059413 PMC4982045

[12] Yuan Y , Yuan L , Li L , et al. Mitochondrial transfer from mesenchymal stem cells to macrophages restricts inflammation and alleviates kidney injury in diabetic nephropathy mice via PGC‐1α activation. Stem Cells. 2021;39(7):913‐928. doi:10.1002/STEM.3375 33739541

[13] Piekarska K , Urban‐Wójciuk Z , Kurkowiak M , et al. Mesenchymal stem cells transfer mitochondria to allogeneic Tregs in an HLA‐dependent manner improving their immunosuppressive activity. Nat Commun. 2022;13(1):856. doi:10.1038/s41467-022-28338-0 35165293 PMC8844425

[14] Do JS , Zwick D , Kenyon JD , et al. Mesenchymal stromal cell mitochondrial transfer to human induced T‐regulatory cells mediates FOXP3 stability. Sci Rep. 2021;11(1):10676. doi:10.1038/S41598-021-90115-8 34021231 PMC8140113

[15] Court AC , Le‐Gatt A , Luz‐Crawford P , et al. Mitochondrial transfer from MSCs to T cells induces Treg differentiation and restricts inflammatory response. EMBO Rep. 2020;21(2):e48052. doi:10.15252/EMBR.201948052 31984629 PMC7001501

[16] Luz‐Crawford P , Hernandez J , Djouad F , et al. Mesenchymal stem cell repression of Th17 cells is triggered by mitochondrial transfer. Stem Cell Res Ther. 2019;10(1):1‐13. doi:10.1186/S13287-019-1307-9 31370879 PMC6676586

[17] Akhter W , Nakhle J , Vaillant L , et al. Transfer of mesenchymal stem cell mitochondria to CD4+ T cells contributes to repress Th1 differentiation by downregulating T‐bet expression. Stem Cell Res Ther. 2023;14(1):1‐16. doi:10.1186/S13287-022-03219-X 36694226 PMC9875419

[18] Irwin RM , Thomas MA , Fahey MJ , Mayán MD , Smyth JW , Delco ML . Connexin 43 regulates intercellular mitochondrial transfer from human mesenchymal stromal cells to chondrocytes. *bioRxiv* . doi:10.1101/2024.03.18.585552 PMC11468299 39390589

[19] Yarosz EL , Chang CH . The role of reactive oxygen species in regulating T cell‐mediated immunity and disease. Immune Netw. 2018;18(1):e14. doi:10.4110/IN.2018.18.E14 29503744 PMC5833121

[20] Zhang H , Wang L , Chu Y . Reactive oxygen species: the signal regulator of B cell. Free Radic Biol Med. 2019;142:16‐22. doi:10.1016/J.FREERADBIOMED.2019.06.004 31185253

[21] Han D , Zheng X , Wang X , Jin T , Cui L , Chen Z . Mesenchymal stem/stromal cell‐mediated mitochondrial transfer and the therapeutic potential in treatment of neurological diseases. Stem Cells Int. 2020;2020:8838046. doi:10.1155/2020/8838046 32724315 PMC7364205

[22] Vallabhaneni KC , Haller H , Dumler I . Vascular smooth muscle cells initiate proliferation of mesenchymal stem cells by mitochondrial transfer via tunneling nanotubes. Stem Cells Dev. 2012;21(17):3104‐3113. doi:10.1089/SCD.2011.0691 22676452 PMC3495124

[23] Osteikoetxea‐Molnár A , Szabó‐Meleg E , Tóth EA , et al. The growth determinants and transport properties of tunneling nanotube networks between B lymphocytes. Cell Mol Life Sci. 2016;73(23):4531‐4545. doi:10.1007/S00018-016-2233-Y 27125884 PMC11108537

[24] Zamora R , Korff S , Mi Q , et al. A computational analysis of dynamic, multi‐organ inflammatory crosstalk induced by endotoxin in mice. PLoS Comput Biol. 2018;14(11):e1006582. doi:10.1371/JOURNAL.PCBI.1006582 30399158 PMC6239343

[25] Zhang Z , Wang L , Li F , et al. Therapeutic effects of human umbilical cord mesenchymal stem cell on sepsis‐associated encephalopathy in mice by regulating PI3K/AKT pathway. J Integr Neurosci. 2022;21(1):38. doi:10.31083/J.JIN2101038/1757-448X-21-1-038.PDF 35164474

[26] Li H , Dai H , Li J . Immunomodulatory properties of mesenchymal stromal/stem cells: the link with metabolism. J Adv Res. 2023;45:15‐29. doi:10.1016/J.JARE.2022.05.012 35659923 PMC10006530

[27] Xu Y , Shen J , Ran Z . Emerging views of mitophagy in immunity and autoimmune diseases. Autophagy. 2020;16(1):3‐17. doi:10.1080/15548627.2019.1603547 30951392 PMC6984455

[28] Malekpour K , Hazrati A , Soudi S , Hashemi SM . Mechanisms behind therapeutic potentials of mesenchymal stem cell mitochondria transfer/delivery. J Control Release. 2023;354:755‐769. doi:10.1016/J.JCONREL.2023.01.059 36706838

[29] Tan AS , Baty JW , Dong LF , et al. Mitochondrial genome acquisition restores respiratory function and tumorigenic potential of cancer cells without mitochondrial DNA. Cell Metab. 2015;21(1):81‐94. doi:10.1016/J.CMET.2014.12.003 25565207

[30] Esteban‐Martínez L , Sierra‐Filardi E , McGreal RS , et al. Programmed mitophagy is essential for the glycolytic switch during cell differentiation. EMBO J. 2017;36(12):1688‐1706. doi:10.15252/EMBJ.201695916 28465321 PMC5470043

[31] Plotnikov EY , Khryapenkova TG , Galkina SI , Sukhikh GT , Zorov DB . Cytoplasm and organelle transfer between mesenchymal multipotent stromal cells and renal tubular cells in co‐culture. Exp Cell Res. 2010;316(15):2447‐2455. doi:10.1016/J.YEXCR.2010.06.009 20599955

[32] Soto‐Heredero G , de Gómez las Heras MM , Gabandé‐Rodríguez E , Oller J , Mittelbrunn M . Glycolysis—a key player in the inflammatory response. FEBS J. 2020;287(16):3350. doi:10.1111/FEBS.15327 32255251 PMC7496292

[33] Islam A , Shen X , Hiroi T , Moss J , Vaughan M , Levine SJ . The Brefeldin A‐inhibited guanine nucleotide‐exchange protein, BIG2, regulates the constitutive release of TNFR1 exosome‐like vesicles. J Biol Chem. 2007;282(13):9591‐9599. doi:10.1074/JBC.M607122200 17276987

[34] Nylander S , Kalies I . Brefeldin a, but not monensin, completely blocks CD69 expression on mouse lymphocytes:: efficacy of inhibitors of protein secretion in protocols for intracellular cytokine staining by flow cytometry. J Immunol Methods. 1999;224(1–2):69‐76. doi:10.1016/S0022-1759(99)00010-1 10357208

[35] Morris RL , Hollenbeck PJ . The regulation of bidirectional mitochondrial transport is coordinated with axonal outgrowth. J Cell Sci. 1993;104(3):917‐927. doi:10.1242/JCS.104.3.917 8314882

[36] Pålsson‐McDermott EM , O'Neill LAJ . Targeting immunometabolism as an anti‐inflammatory strategy. Cell Res. 2020;30(4):300‐314. doi:10.1038/s41422-020-0291-z 32132672 PMC7118080

[37] Konari N , Nagaishi K , Kikuchi S , Fujimiya M . Mitochondria transfer from mesenchymal stem cells structurally and functionally repairs renal proximal tubular epithelial cells in diabetic nephropathy in vivo. Sci Rep. 2019;9(1):5184. doi:10.1038/S41598-019-40163-Y 30914727 PMC6435708

[38] Han H , Hu J , Yan Q , et al. Bone marrow‐derived mesenchymal stem cells rescue injured H9c2 cells via transferring intact mitochondria through tunneling nanotubes in an in vitro simulated ischemia/reperfusion model. Mol Med Rep. 2016;13(2):1517‐1524. doi:10.3892/MMR.2015.4726 26718099 PMC4732861

[39] Babenko VA , Silachev DN , Popkov VA , et al. Miro1 enhances mitochondria transfer from multipotent mesenchymal stem cells (MMSC) to neural cells and improves the efficacy of cell recovery. Molecules. 2018;23(3):687. doi:10.3390/MOLECULES23030687 29562677 PMC6017474

[40] Islam MN , Das SR , Emin MT , et al. Mitochondrial transfer from bone‐marrow‐derived stromal cells to pulmonary alveoli protects against acute lung injury. Nat Med. 2012;18(5):759‐765. doi:10.1038/nm.2736 22504485 PMC3727429

[41] Huh JW , Kim WY , Park YY , et al. Anti‐inflammatory role of mesenchymal stem cells in an acute lung injury mouse model. Acute and Critical Care. 2018;33(3):154‐161. doi:10.4266/ACC.2018.00619 31723879 PMC6786701

[42] Feng YW , Wu C , Liang FY , et al. hUCMSCs mitigate LPS‐induced trained immunity in ischemic stroke. Front Immunol. 2020;11:1746. doi:10.3389/FIMMU.2020.01746 33013828 PMC7516337

[43] Hwang JW , Lee MJ , Chung TN , et al. The immune modulatory effects of mitochondrial transplantation on cecal slurry model in rat. Crit Care. 2021;25(1):20. doi:10.1186/S13054-020-03436-X 33413559 PMC7789332

[44] Barrasso AP , Tong X , Poché RA . The mito::mKate2 mouse: a far‐red fluorescent reporter mouse line for tracking mitochondrial dynamics in vivo. Genesis. 2018;56(2):e23087. doi:10.1002/DVG.23087 PMC5818295 29243279

[45] Hajkova M , Javorkova E , Zajicova A , Trosan P , Holan V , Krulova M . A local application of mesenchymal stem cells and cyclosporine a attenuates immune response by a switch in macrophage phenotype. J Tissue Eng Regen Med. 2017;11(5):1456‐1465. doi:10.1002/TERM.2044 26118469

[46] Hajkova M , Hermankova B , Javorkova E , et al. Mesenchymal stem cells attenuate the adverse effects of immunosuppressive drugs on distinct T cell subopulations. Stem Cell Rev Rep. 2017;13(1):104‐115. doi:10.1007/S12015-016-9703-3 27866327

[47] Marvanova A , Kasik P , Elsnicova B , et al. Continuous short‐term acclimation to moderate cold elicits cardioprotection in rats, and alters β‐adrenergic signaling and immune status. Sci Rep. 2023;13(1):18287. doi:10.1038/S41598-023-44205-4 37880253 PMC10600221

[48] Kim D , Langmead B , Salzberg SL . HISAT: a fast spliced aligner with low memory requirements. Nat Methods. 2015;12(4):357‐360. doi:10.1038/NMETH.3317 25751142 PMC4655817

[49] Danecek P , Bonfield JK , Liddle J , et al. Twelve years of SAMtools and BCFtools. Gigascience. 2021;10(2):1‐4. doi:10.1093/GIGASCIENCE/GIAB008 PMC7931819 33590861

[50] Li H , Handsaker B , Wysoker A , et al. The sequence alignment/map format and SAMtools. Bioinformatics. 2009;25(16):2078‐2079. doi:10.1093/BIOINFORMATICS/BTP352 19505943 PMC2723002

[51] Kleverov M , Zenkova D , Kamenev V , Sablina M , Artyomov MN , Sergushichev AA . Phantasus: web‐application for visual and interactive gene expression analysis. *bioRxiv* . doi:10.1101/2022.12.10.519861 PMC11147506 38742735

