# Supplementary material for: Mesenchymal stem cell‐mediated mitochondrial transfer regulates the fate of B lymphocytes
Source: Eur J Clin Invest. 2025 May 15;55(10):e70073. doi: 10.1111/eci.70073 (PMC12434451; doi:10.1111/eci.70073)
Supplement: Supplementary file 1 — Figure S1: Gating strategy of mitochondrial transfer to CD45+CD19+ or CD45+Ly6G+ immune cells. Gating strategy: debris was excluded based on forward and side scatter, then doublets were excluded, and immune cells were gated based on CD45 positivity on Hoechst 33258 negative population (live). (A) Splenocytes isolated from BALB/c mice were analysed to determine mitochondrial transfer after 3 h co‐culture with MSCs labelled with MitoTracker Red CMXRos by flow cytometry. From CD45+ cells, B cells were gated as CD19+ cells. MitoTracker Red positivity on CD19+ cells (CD19+MTR+) was determined based on the FMO control. (B) Peritoneal cells isolated from BALB/c mice were analysed to determine Ly6G+ neutrophile, granulocyte and monocyte infiltration. Figure S2: Gating strategy of TNF‐α stained as intracellular protein. Debris was excluded based on forward and side scatter, then doublets were excluded, and immune cells were gated based on CD45 positivity on LIVE/DEAD Fixable violet negative population (live). From CD45+ cells, B cells were gated as CD19+ cells. Acceptors and nonacceptors of mitochondria were gated as mKate+ and mKate− cells. From both these populations TNF‐α positive cells were determined. Figure S3: Mitochondrial transfer in a contactless co‐culture system. MitoTracker Red‐stained MSCs were co‐cultured with immune cells in a double chamber TransWell system. Cells were separated by a membrane with 3 μm pores. Ctrl – cells co‐cultured in a standard single chamber system. TW – cells co‐cultured in a double chamber TransWell system. Data are presented as mean ± SD of three independent experiments (n = 3), where n corresponds to the number of mice used. Statistically significant differences between groups are indicated by asterisks (*p < .05, **p < .01 and ***p < .001). Figure S4: The effect of ROS production and mitophagy on mitochondrial transfer. (A) ROS production by different immune cell populations, shown as the mean fluorescence intensity (MFI) of the ROS [file ECI-55-e70073-s001.docx]

**
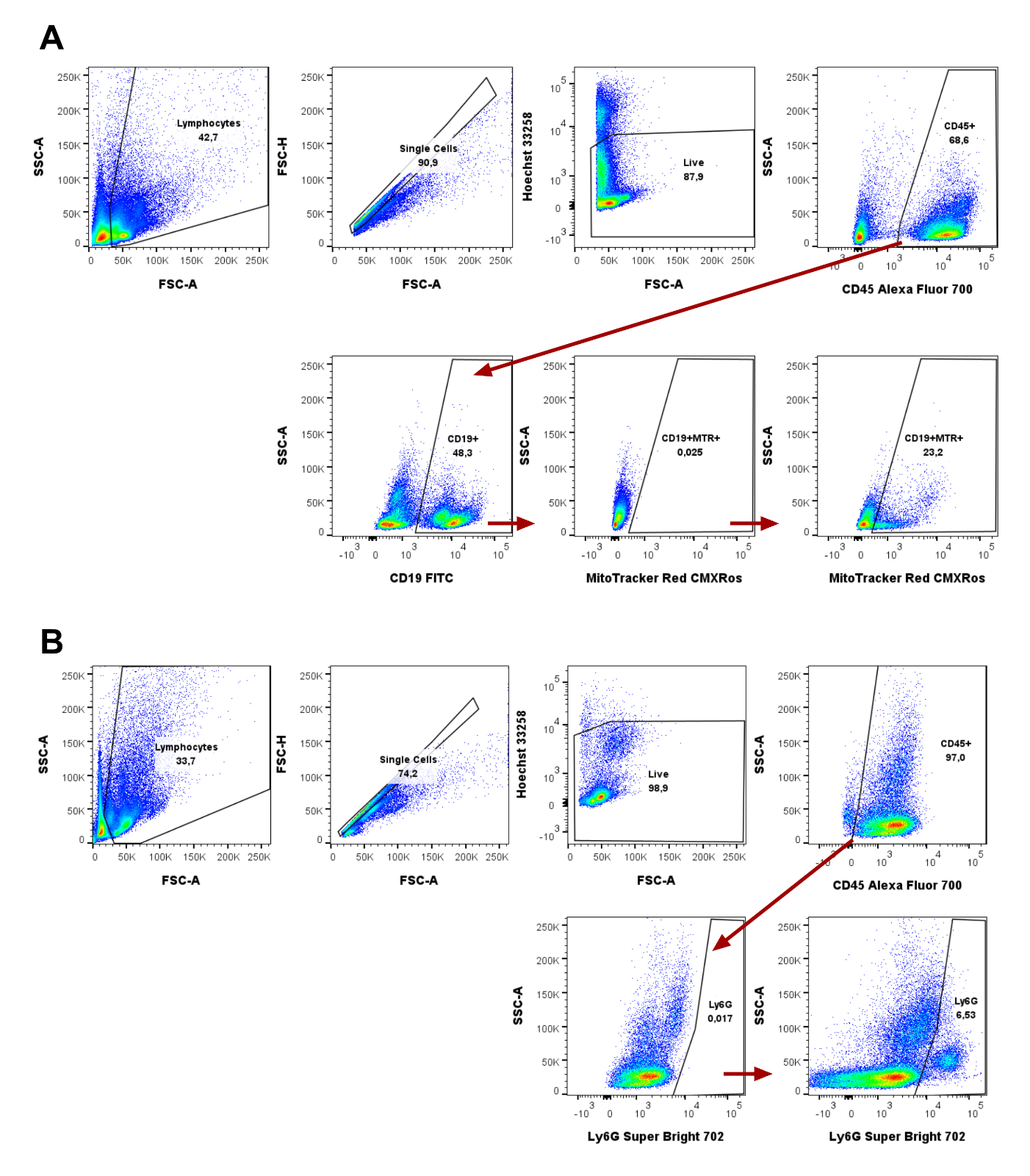
**

**Figure S1**: Gating strategy of mitochondrial transfer to CD45^+^CD19^+^ or CD45^+^Ly6G^+^ immune cells. Gating strategy: debris was excluded based on forward and side scatter, then doublets were excluded, and immune cells were gated based on CD45 positivity on Hoechst 33258 negative population (live). **A** Splenocytes isolated from BALB/c mice were analyzed to determine mitochondrial transfer after 3h co-culture with MSCs labeled with MitoTracker Red CMXRos by flow cytometry. From CD45^+^ cells, B cells were gated as CD19^+^ cells. MitoTracker Red positivity on CD19^+^ cells (CD19^+^MTR^+^) was determined based on the FMO control. **B** Peritoneal cells isolated from BALB/c mice were analyzed to determine Ly6G^+^ neutrophile, granulocyte and monocyte infiltration.


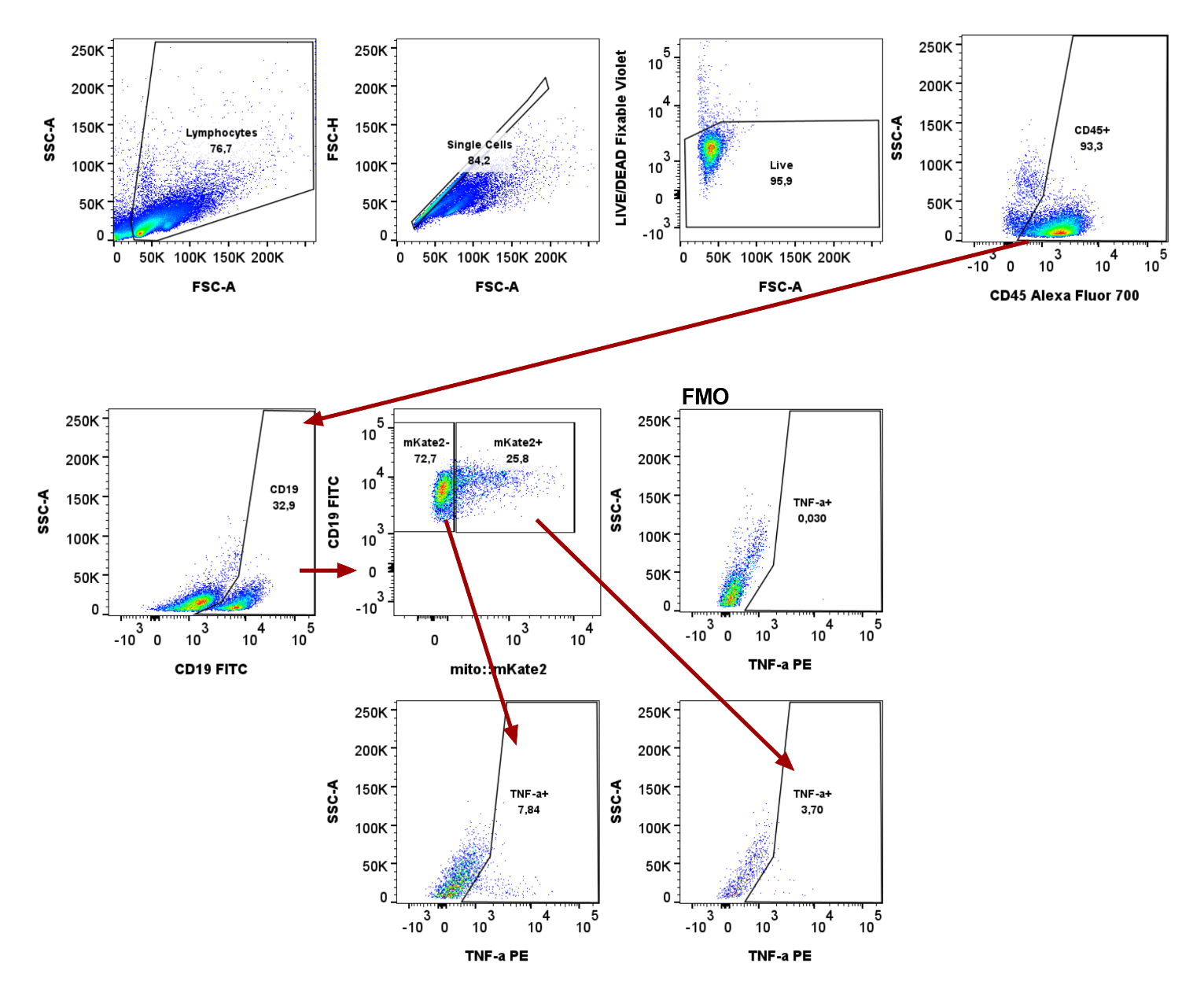


**Figure S2:** Gating strategy of TNF-α stained as intracellular protein. Debris was excluded based on forward and side scatter, then doublets were excluded, and immune cells were gated based on CD45 positivity on LIVE/DEAD Fixable violet negative population (live). From CD45^+^ cells, B cells were gated as CD19^+^ cells. Acceptors and non-acceptors of mitochondria were gated as mKate^+^ and mKate^-^ cells. From both these populations TNF-α positive cells were determined.


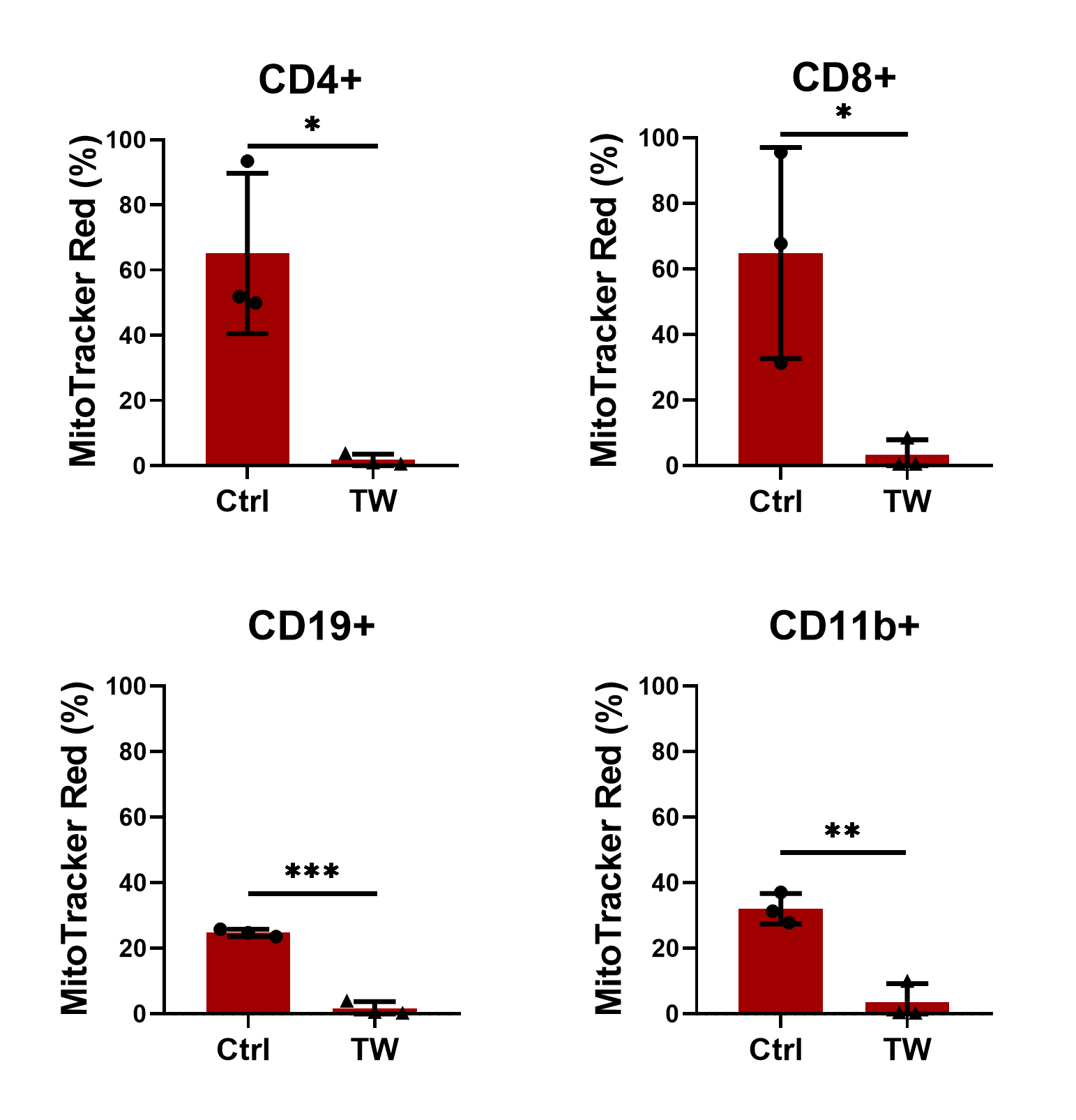


**Figure S3:** Mitochondrial transfer in a contactless co-culture system. MitoTracker Red-stained MSCs were co-cultured with immune cells in a double chamber TransWell system. Cells were separated by a membrane with 3 µm pores. Ctrl – cells co-cultured in a standard single chamber system. TW – cells co-cultured in a double chamber TransWell system. Data are presented as mean ± SD of three independent experiments (n = 3), where *n* corresponds to the number of mice used. Statistically significant differences between groups are indicated by asterisks (**P*<0.05, ***P<*0.01, ****P<*0.001).


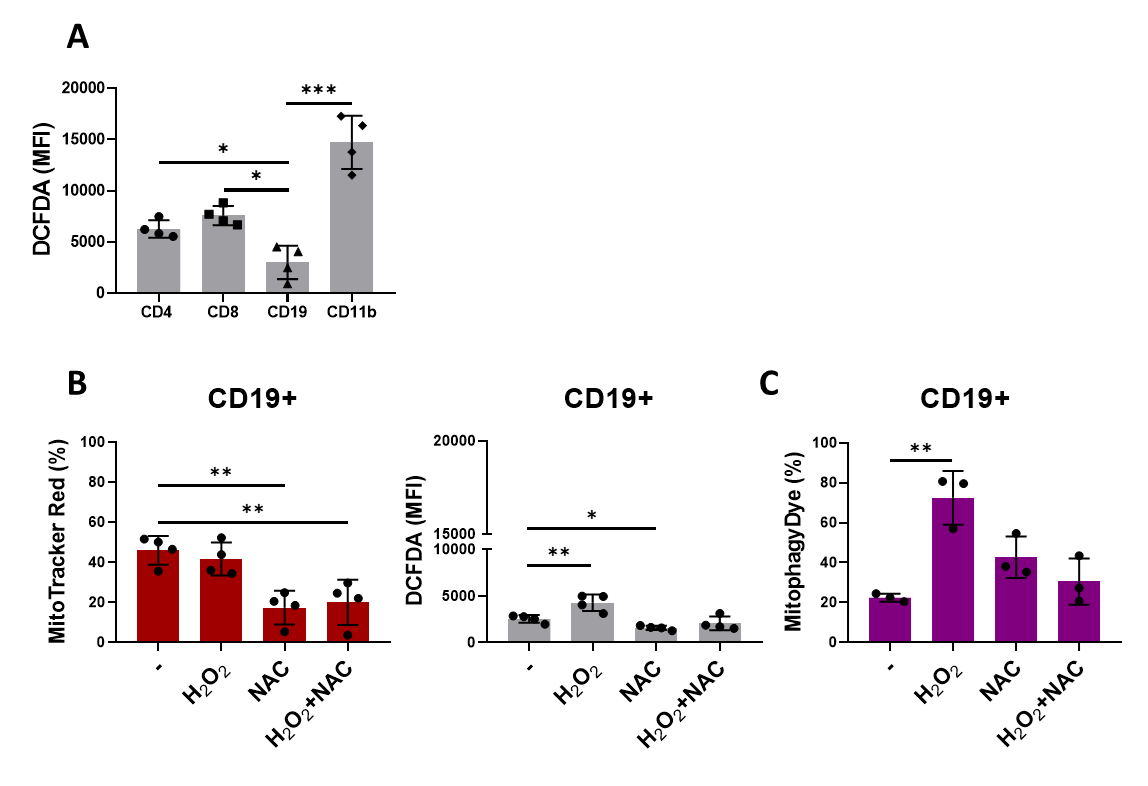


**Figure S4:** The effect of ROS production and mitophagy on mitochondrial transfer. **A** ROS production by different immune cell populations, shown as the mean fluorescence intensity (MFI) of the ROS marker DCFDA. Data are presented as mean ± SD of four independent experiments. **B** MSC-derived mitochondrial transfer and corresponding ROS production by CD19^+^ immune cells, shown as a percentage of MitoTracker Red positive cells or MFI of ROS marker DCFDA. Prior to co-culture with MSCs, immune cells were cultured for 24 h either untreated or in the presence of 5 µM hydrogen peroxide, 5 mM NAC, or a combination of both. Data are presented as mean ± SD of four independent experiments (n = 4), where *n* corresponds to the number of mice used. **C** Mitophagy in CD19^+^ immune cells which were cultured as mentioned previously, shown as a percentage of MitophagyDye positive cells. Data are presented as mean ± SD of three independent experiments (n = 3), where *n* corresponds to the number of mice used. In all figures, statistically significant differences between groups are indicated by asterisks (**P*<0.05, ***P<*0.01, ****P<*0.001).

**B**

**A**

**Figure S5:** The effect of mitochondrial transfer on CD19^+^ cells. **A** Survival rate cells that received mitochondria (mit^pos^) or did not receive mitochondria (mit^neg^) CD19^+^ cells cultured for 24 h after FACS-sorting, shown as counts of living cells. **B** Analysis of metabolic activity of mit^neg^ and mit^pos^ CD19^+^ cells using the WST-1 assay 24 h after FACS-sorting. Data are presented as mean ± SD of three independent experiments (n = 3), where *n* corresponds to the number of mice used. Statistically significant differences between groups are indicated by asterisks (**P*<0.05, ***P<*0.01, ****P<*0.001).


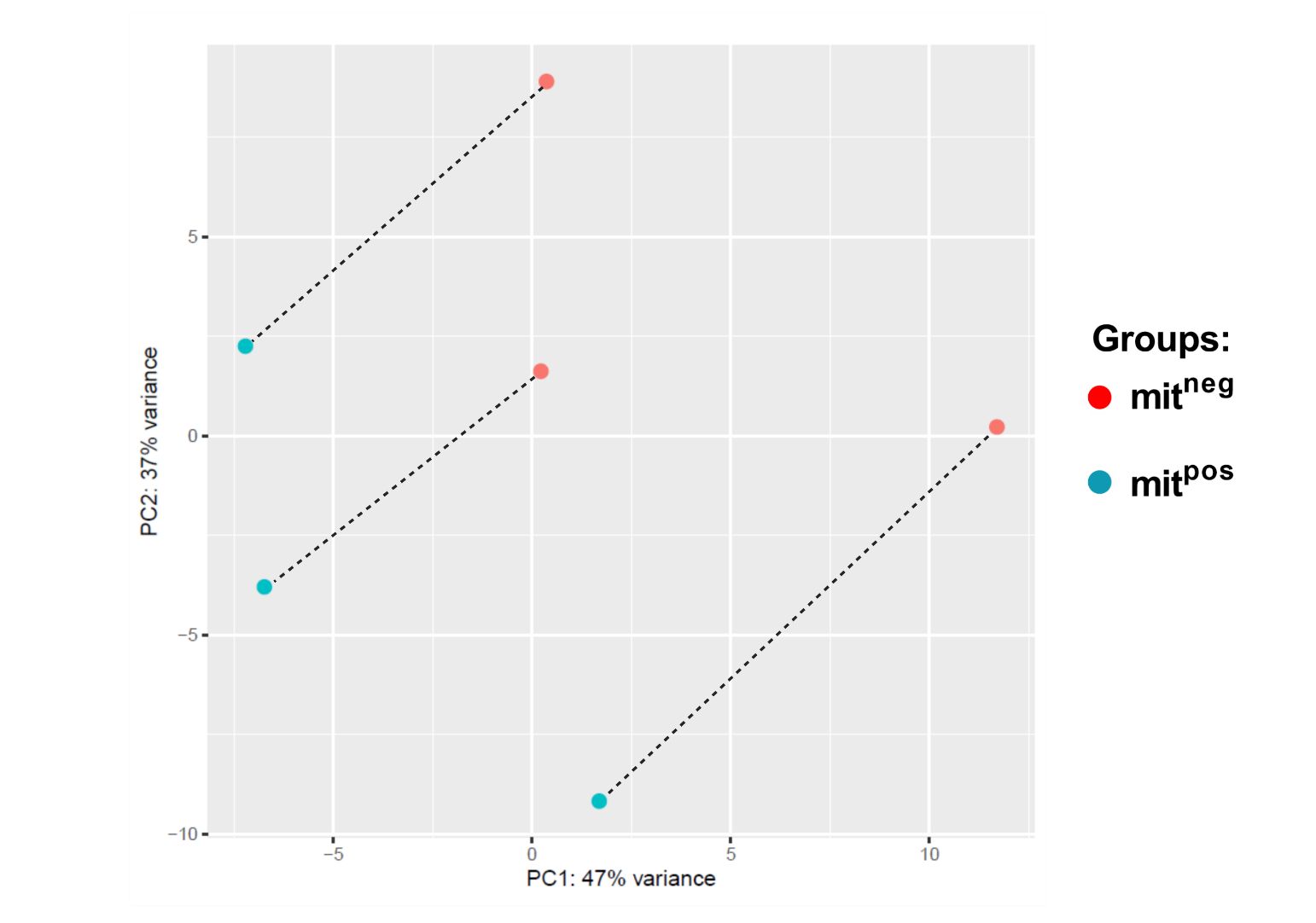


**Figure S6:** Principal component analysis (PCA) plot of normalized gene counts. Principal component (PC) 1 (red) vs PC2 (blue) colored by means of mitochondria acquirement. Mit^neg^ and mit^pos^ samples coming from the same mouse are connected with dotted lines.


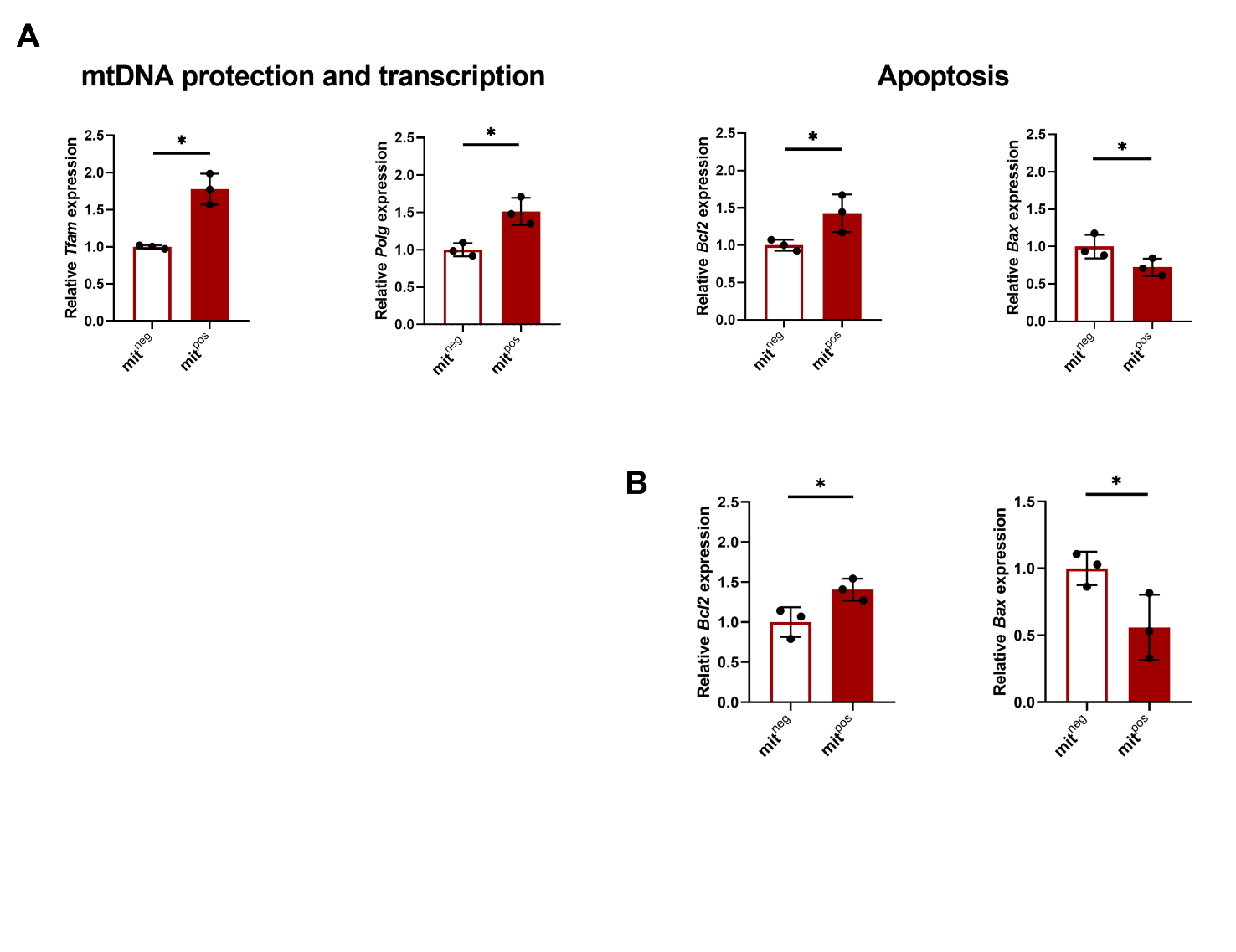


**Figure S7: Protective and pro-survival effect of mitochondrial transfer. A** Relative expression of genes *Tfam* and *Polg*, which are associated with mtDNA protection and transcription, as well as apoptosis-related genes *Bcl2* and *Bax* obtained from the RNA-sequencing data. **B** Relative expression of genes *Bcl2* and *Bax* obtained from qPCR analysis. Data are presented as mean ± SD of three independent experiments. (n = 3), where n corresponds to the number of mice used. Statistically significant differences between groups are indicated by asterisks (**P*<0.05, ***P<*0.01, ****P<*0.001).
